# Exploring short k-mer profiles in cells and mobile elements from *Archaea* highlights the major influence of both the ecological niche and evolutionary history

**DOI:** 10.1186/s12864-021-07471-y

**Published:** 2021-03-16

**Authors:** Ariane Bize, Cédric Midoux, Mahendra Mariadassou, Sophie Schbath, Patrick Forterre, Violette Da Cunha

**Affiliations:** 1 Université Paris-Saclay, INRAE, PROSE, F-92761 Antony, France; 2 Université Paris-Saclay, INRAE, MaIAGE, F-78350 Jouy-en-Josas, France; 3 Université Paris-Saclay, INRAE, BioinfOmics, MIGALE bioinformatics facility, F-78350 Jouy-en-Josas, France; 4 Institut Pasteur, Unité de Virologie des Archées, Département de Microbiologie, 25 Rue du Docteur Roux, 75015 Paris, France; 5 Université Paris-Saclay, CEA, CNRS, Institute for Integrative Biology of the Cell (I2BC), 91198 Gif-sur-Yvette, France

**Keywords:** Extrachromosomal element, Virus, Plasmid, 5-mer, Codon composition, Multivariate analysis, Signature, Halophily, Hyperthermophily, Host transfer

## Abstract

**Background:**

K-mer-based methods have greatly advanced in recent years, largely driven by the realization of their biological significance and by the advent of next-generation sequencing. Their speed and their independence from the annotation process are major advantages. Their utility in the study of the mobilome has recently emerged and they seem a priori adapted to the patchy gene distribution and the lack of universal marker genes of viruses and plasmids.

To provide a framework for the interpretation of results from k-mer based methods applied to archaea or their mobilome, we analyzed the 5-mer DNA profiles of close to 600 archaeal cells, viruses and plasmids. *Archaea* is one of the three domains of life. Archaea seem enriched in extremophiles and are associated with a high diversity of viral and plasmid families, many of which are specific to this domain. We explored the dataset structure by multivariate and statistical analyses, seeking to identify the underlying factors.

**Results:**

For cells, the 5-mer profiles were inconsistent with the phylogeny of archaea. At a finer taxonomic level, the influence of the taxonomy and the environmental constraints on 5-mer profiles was very strong. These two factors were interdependent to a significant extent, and the respective weights of their contributions varied according to the clade. A convergent adaptation was observed for the class *Halobacteria*, for which a strong 5-mer signature was identified. For mobile elements, coevolution with the host had a clear influence on their 5-mer profile. This enabled us to identify one previously known and one new case of recent host transfer based on the atypical composition of the mobile elements involved. Beyond the effect of coevolution, extrachromosomal elements strikingly retain the specific imprint of their own viral or plasmid taxonomic family in their 5-mer profile.

**Conclusion:**

This specific imprint confirms that the evolution of extrachromosomal elements is driven by multiple parameters and is not restricted to host adaptation. In addition, we detected only recent host transfer events, suggesting the fast evolution of short k-mer profiles. This calls for caution when using k-mers for host prediction, metagenomic binning or phylogenetic reconstruction.

**Supplementary Information:**

The online version contains supplementary material available at 10.1186/s12864-021-07471-y.

## Background

In the field of nucleic acid sequence analysis, k-mer based methods have greatly advanced in recent years, supported by the advent of next-generation sequencing (reviewed in [1]). As the main advantages, they usually provide reasonable computation durations compared to most traditional alignment-based tools; they are also annotation-independent, and they enable the comparison of incomplete or nonhomologous sequences on a common basis. While they first emerged for practical purposes, their biological significance was subsequently established (reviewed in [2]). In particular, it appeared that the composition of short k-mers is conserved throughout the genome sequence, giving rise to the concept of a k-mer signature, originally based on dinucleotide composition [3]. This finding raised questions regarding the evolutionary significance of this concept and of the underlying mechanisms [4]. Meanwhile, a variety of k-mer-based applications started to proliferate. In the field of environmental microbiology, many k-mer-based tools are dedicated to metagenomic analysis. The k-mer composition of contigs can be used for binning, an important step in the reconstruction of metagenome-assembled genomes (MAGs) (e.g. [5, 6]). It is also used for the taxonomic assignation of sequences (e.g. [7–9]) and to compare different metagenomes by examining distances between k-mer profiles (e.g. [10, 11]). Quite recently, tools specifically dedicated to mobile elements have been developed, that seem a priori adapted to the patchy gene distribution and to the lack of universal marker genes of viruses and plasmids. They enable, for instance, the prediction of viral [12] or plasmid [13] sequences from metagenomes, the assignment of hosts to viruses [14] or plasmids [13], or the classification of viruses [15]. For the study of microbial diversity and evolution, the possibility of using k-mers for phylogenetic [16–19] or evolutionary network [20, 21] reconstruction is also being explored; its application to the detection of horizontal gene transfer (HGT) was proposed more than 10 years ago [22], and a tool for HGT detection within metagenomic data has been recently published [23].

Since these tools are generally based on statistical methods, the results may inevitably contain false or true positives. It is thus necessary to continue exploring k-mer signatures across the genomosphere to establish a framework for interpretation of results obtained with k-mer-based tools. In the present work, we focused specifically on the cells and mobile elements from *Archaea*, one of the three domains of life.

The diversity of viruses and plasmids in *Archaea* is high, with a great number of approved families compared to the relatively low number of isolated elements [24–26]. This provides an interesting case for comparing k-mer composition among hosts and viruses. In particular, viruses of extreme thermophilic crenarchaea are highly diverse. They often belong to *Archaea*-specific viral families, with unusual morphotypes. In the class *Halobacteria,* head-and-tail viruses belonging to *Caudovirales* are abundant and are predominant in hypersaline environments, which are dominated by haloarchaea [27]. While *Caudovirales* is a cosmopolitan order of viruses (the most abundant order infecting *Bacteria* [28]), *Halobacteria* members are also infected by *Archaea*-specific viral families, such as *Pleioipoviridae*. Many archaeal plasmids have not yet been classified into well-defined families; however, several families of plasmids have been defined according to plasmid size, replication mode, and genomic content (reviewed in [25]).

Among archaea, there are no known pathogens for humans, plants or animals, so there is no overrepresentation bias linked to pathogens in the databases. Other biases are, however, present: the mobile elements from several archaeal taxonomic groups (orders or even phyla,) are very poorly represented in public databases, so the view on global diversity remains incomplete. In addition to the diversity of their mobile elements, archaea constitute an interesting case in terms of adaptation or loss of adaptation to extreme environments, which has played an important role in their evolutionary history [29].

Several studies on k-mer signatures previously included archaeal genomes. For instance, in 1999, Campbell et al. [30] studied genome signatures across a wide phylogenetic range, encompassing bacteria, archaea, plasmids and mitochondrial DNA. This work highlighted the similarity of signatures between hosts and plasmids, the lack of consistent signatures among thermophiles and, finally, the high signature divergence among five archaeal genomes available at that time. In 2006, van Passel et al. [31] showed the difference in dinucleotide composition between hosts and plasmids in *Archaea* and *Bacteria*. In 2008, Bohlin et al. [32] obtained a similar trend by using 4-mers and zero-order Markov models. The same authors studied the composition of bacterial and archaeal genomes in 2- to 8-mers, with 44 archaeal genomes among the 581 analyzed genomes. They observed a higher variability in AT-rich and host-associated genomes compared to GC rich or free-living archaea and bacteria [33].

Currently, the number of publicly available genomes has greatly increased, warranting a new study of signatures across the domain *Archaea*. Selecting close to 600 cellular, viral and plasmid genomes, we applied metrics based on short k-mer profiles to understand how mobile elements are distributed with respect to their hosts in the profile landscape. We used multivariate and statistical analyses to explore the dataset structure and identify some key structuring factors, namely, the taxonomic classification, the genomic GC content, the ecological niche and, for mobile elements, the taxonomy of the host. Moreover, we examined whether 5-mer profiles enable the detection of singular evolutionary trajectories, such as host transfers, among mobile elements. We also searched for 5-mer signatures for halophily and hyperthermophily in *Archaea*.

## Results

### The 5-mer profiles of archaeal genomes are influenced by the taxonomy and GC content

Before focusing on extrachromosomal elements, we first analyzed the 5-mer profile distribution of archaeal cellular genomes. We selected 239 archaeal genomes, focusing mainly on taxonomic groups for which many plasmids and/or viruses have already been classified into distinct families: *Halobacteria*, *Sulfolobales*, *Thermococcales* and a few other groups of *Euryarchaeota* and *Crenarchaeota*.

**Fig. 1:**
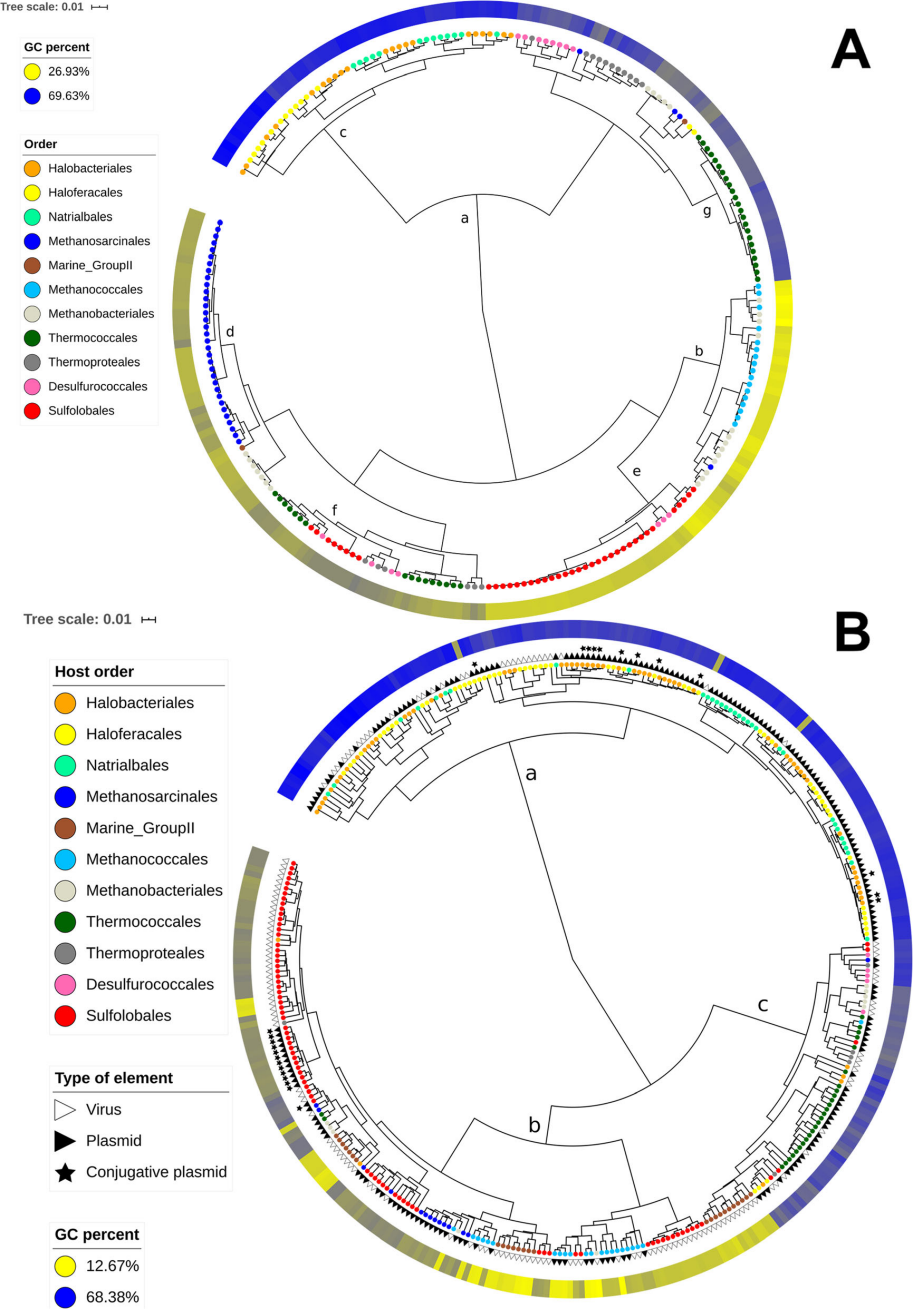
Dendrograms based on 5-mer frequencies for archaeal cells and mobile elements. **a**. Archaeal cells. **b**. Archaeal viruses and plasmids

We first noticed from the dendrogram obtained by hierarchical clustering that the sequences were distributed into two main clusters according to GC content values, suggesting a major influence of the GC content on the k-mer distribution (Fig. 1a). The most GC-rich cluster (Fig. 1a, letter c) exclusively included *Halobacteria* members, consistent with the fact that *Halobacteria* have a high genomic GC-content, 63.28% ± 4.29 SD on average in our dataset. At the other extreme, the less GC-rich cluster (Fig. 1a, letter b) comprised only Group I methanogens (*Methanococcales* and *Methanobacteriales*), except for one Group II *Methanosarcinales* genome.

We also identified taxonomy as an important factor, and many clusters were dominated by a single taxonomic group (Fig. 1a). In particular, all members of the class *Halobacteria* were located in a single cluster (Fig. 1a, letters c) with only two exceptions, corresponding to the two *Haloquadratum walsbyi* genomes (order *Haloferacales*). Similarly, 33 out of 37 members of the order *Methanosarcinales* were gathered in a single cluster (Fig. 1a, letter d). Members of the order *Sulfolobales* were divided into a major cluster (31 genomes out of 39) and a minor cluster (8 genomes out of 39) (Fig. 1a, letters e and f, respectively). The latter corresponded to the *Metallosphaera* genomes, which have a higher GC content than the other *Sulfolobales* genomes. The 17 members of the order *Methanococcales* were divided into two neighboingr clusters (Fig. 1a, within cluster b), which also included several *Methanobacteriales* members, which are Group I methanogens, similar to *Methanococcales* members.

We did not observe similar clustering for *Methanobacteriales*, *Thermococcales*, *Thermoproteales* and *Desulfurococcales.* In such cases, archaea belonging to the same order were distributed into several clusters, sometimes distant across the dendrogram. However, at the local scale, small- to medium-sized clusters enriched in one of these orders were still visible, such as a medium-sized cluster comprising exclusively *Thermococcales* members (23 genomes out of 39) (Fig. 1a, letter g).

To quantify the relative contribution of the taxonomy and of the GC content to the 5-mer composition, we performed a permutational multivariate analysis of variance (PERMANOVA) (Additional file 1). We applied PERMANOVA to the pairwise Euclidian distance matrix computed from the 5-mer profiles, which we will denote as D_5_cells_ hereafter. Among the three considered taxonomic levels (phylum, order, genus), order had the strongest influence; it alone explained 75.94% of the cell profile dissimilarity variance (model: D_5_cells_ ~ Genus), compared to 7.06% for phylum (D_5_cells_ ~ Phylum) and 17.74% for genus, when the effect of the phylum and order was first removed (D_5_cells_ ~ Phylum*Order*Genus).

Notably, the GC content alone contributed almost as much to the variance (69.10%, D_5_cells_ ~ GC%) as the taxonomic rank of the order (D_5_cells_ ~ order). These last two factors appeared to be highly dependent, explaining 56.71% of the cell dissimilarity variance (D_5_cells_ ~ order*GC%) in an indistinguishable manner.

Despite the strong influence of the taxonomy, the global topology of the dendrogram obtained by hierarchical clustering was inconsistent with the phylogeny of archaea. While *Sulfolobales* belongs to the *Crenarchaeota* phylum, its main cluster grouped with a cluster dominated by Group I methanogens from the *Euryarchaeota* phylum. Moreover, within the major *Halobacteria* cluster, archaea from the three orders *Haloferacales*, *Halobacteriales* and *Natrialbales* were interconnected (especially due to *Halobacteriales*), showing the blurring of phylogenetic information.

### A strong link between the ecological niche and the 5-mer composition of archaeal cellular genomes

Many archaea thrive in extreme conditions, and adaptation to such specific environments has played an important role in their evolution [34, 35]. We therefore assumed that major properties of the environmental niches could be another important factor underlying the 5-mer composition among archaea. We focused on salinity and temperature and defined 8 “Niche” categories. All *Halobacteria* members were categorized as “halophile”. The remaining archaea were labeled according to 7 qualitative growth temperature categories, ranging from “weak mesophile” to “extreme hyperthermophile” (Additional File 2), based on the BacDive database [36] and on the literature, e.g. [37].

**Fig. 2:**
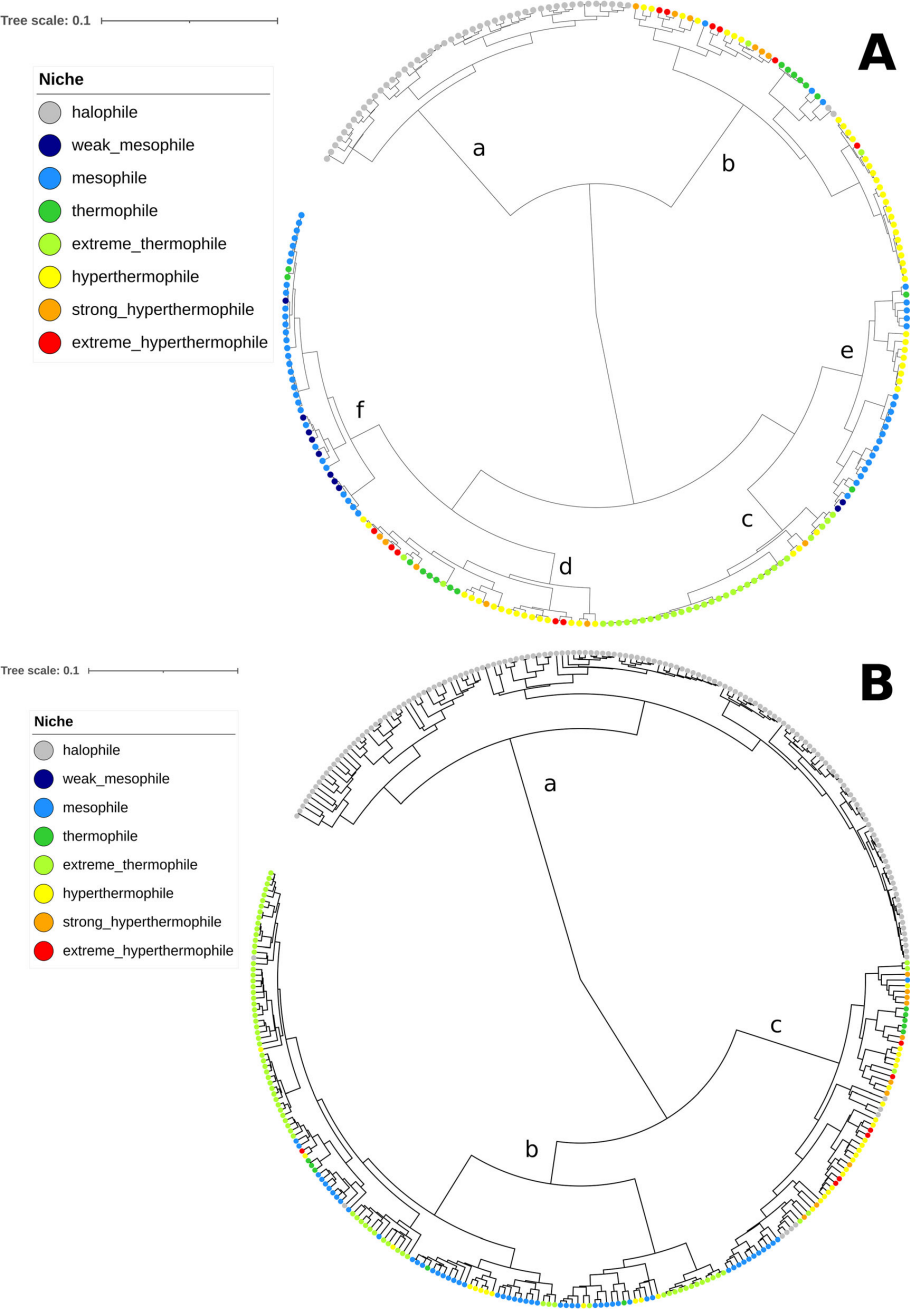
Mapping of temperature and salinity-related growth conditions on the archaeal cell and mobile element dendrograms. **a**. Archaeal cells. **b**. Archaeal viruses and plasmids

The clustering pattern was clearly influenced by the “Niche” categories (Fig. 2 a). Among the 6 main clusters of the dendrogram for cells (Fig. 2 a, clusters a to f), cluster b was largely dominated by thermophiles to extreme hyperthermophiles. Cluster c was dominated by extreme thermophiles, corresponding mostly to *Sulfolobales* members. Cluster d comprised exclusively thermophiles to extreme hyperthermophiles. Finally, clusters e and f were dominated by weak mesophiles and mesophiles, although a small patch of hyperthermophiles was visible in cluster e. *Sulfolobales* comprises exclusively acidophilic members, which could explain their specific signature compared to other thermophilic/hyperthermophilic extrachromosomal elements. Indeed, cytoplasmic pH regulation does not fully compensate for the decrease in intracellular pH in acidic environments: the intracellular pH in acidophiles is higher by approximately 3 to 4 points than that of the surrounding acidic environment, but on the whole, it is still lower than that in neutrophiles [38]. It has previously been suggested that acidophilic archaea and bacteria have purine-poor codons in their long genes [39]; however, the effects of acidophily on compositional features seem to have been studied less than the adaptation to high temperatures.

Based on PERMANOVA, the “Niche” categories explained 64.17% of the dataset variance (D_5_cells_ ~ Niche). Although this percentage is lower than that explained by the taxonomic rank of order (namely, 75.94%), it is still very high. As anticipated, the GC content, taxonomic rank and “Niche” had a high level of dependency (Additional file 1, D_5_cells_ ~ Niche*Order*GC%). In particular, the last two factors explained 60.56% of the cell profile dissimilarity variance in an indistinguishable manner (D_5_cells_ ~ Order*Niche), consistent with the strong links between the ecological niche and the evolutionary history in *Archaea*. Finally, we noticed that a model combining the genomic GC content, ecological niche and taxonomy (order rank) explained almost all the cell dataset variance, namely, 95.48% (Additional file 1, D_5_cells_ ~ Niche*Order*GC%). Overall, a limited number of factors are therefore sufficient to explain the differences in 5-mer composition of the archaeal cell genomes included in our study.

### The extrachromosomal element profiles are also influenced by the GC content and host taxonomy, with higher profile dispersion

We analyzed the 5-mer composition of archaeal plasmids and viruses (extrachromosomal elements) with a similar approach. The obtained dendrogram was divided into two major clusters. One of them (Fig. 1b, letter a), corresponded to elements with the highest GC contents, including nearly all 154 *Halobacteria* mobile elements, except for 9. The second cluster, with the lowest GC content, was divided into two subclusters (Fig. 1b, letters b and c). Subcluster b was dominated by *Sulfolobales* extrachromosomal elements but also included a significant number of extrachromosomal elements from *Methanococcales*, *Methanosarcinales* and *Marine Group II*. Subcluster c was dominated by *Thermococcales* extrachromosomal elements but also comprised significant numbers of extrachromosomal elements from *Marine Group II*, *Desulfurococcales*, *Thermoproteales* and *Methanobacteriales*.

Compared to the pattern obtained for cells, visual inspection showed that the extrachromosomal elements, categorized according to the taxonomy of their host, had a more intertwined distribution, except for viruses and plasmids of *Halobacteria*. Consistent with this observation, the taxonomy of the host at the order level explained only 57.36% of the extrachromosomal element dissimilarity variance (Additional File 3, D_5_mobile_ ~ Host order), compared to 75.94% for the cells. As in the case of cellular genomes, the rank of their hosts appeared more informative at the order level than at the phylum or genus level (Additional File 3, D_5_mobile_ ~ Host Phylum*Host Order*Host Genus).

The less consistent pattern obtained for extrachromosomal elements compared to cells could theoretically reflect more frequent genetic exchanges between extrachromosomal elements present in hosts belonging to different taxonomic groups. However, this does not seem to be the case. For instance, while several cases of host transfers between *Thermococcales* and *Methanococccales* plasmids have been previously documented [25], *Methanococcales* extrachromosomal elements clustered mostly with those of *Sulfolobales* rather than with those of *Thermococcales* in our analysis. Another hypothesis to explain such a complex pattern for extrachromosomal elements could be the influence of their GC content. Indeed, extrachromosomal element genomes harbor, in many cases, a distinct average GC content compared to their hosts (Additional File 4). We noticed that the extent and even the direction of these shifts in GC content varied greatly according to the host’s taxonomy (at the order level) and to the type of extrachromosomal element (Additional File 4). Since the GC content had a strong global influence on the obtained pattern (45.13% of the variance, Additional File 3, D_5_mobile_ ~ GC%), these shifts in GC content could greatly contribute to the more complex pattern obtained for archaeal extrachromosomal elements compared to that obtained for archaeal cells.

Similar to cells, the host taxonomy (at the order level) and the genomic GC-content were highly interdependent factors for extrachromosomal elements (Additional File 3): 39.71% of the dissimilarity variance was explained indistinguishably by these two factors (D_5_mobile_ ~ Host Order*GC% and D_5_mobile_ ~ GC% * Host Order). Interestingly, the taxonomic classification of viruses and plasmids was by far the most influential factor, alone explaining 68.30% of the extrachromosomal element dissimilarity variance (Additional File 3, D_5_mobile_ ~ Family). This could be due partly to the high number of viral and plasmid families in the dataset (60 compared to only 11 different host orders), which must support a better fit of the model. However, this finding also suggests that individual viral and plasmid families could have a specific 5-mer composition.

The extrachromosomal element family and the taxonomy of their hosts at the order level were strongly dependent, since 51.90% of the extrachromosomal element dissimilarity variance was explained indistinguishably by one of the factors (Additional File 3, D_5_mobile_ ~ Host Order*Family and D_5_mobile_ ~ Family*Host Order). This could reflect the fact that the host range of a given plasmid or viral family is limited. The fact that viruses and plasmids coevolved with their hosts and that they were not frequently transferred to new hosts from other orders could explain this limitation.

### A significant but weaker influence of the ecological niche on the 5-mer composition of archaeal extrachromosomal elements

We used the same “Niche” categories and method to analyze plasmids and viruses of archaea (Fig. 2 b). As already identified above (Fig. 2 b), extrachromosomal elements from halophiles grouped together (cluster a), with a very limited number of exceptions. The viruses and plasmids from extreme thermophiles, corresponding mostly to *Sulfolobales*, tended to group with mesophilic extrachromosomal elements, in cluster b. By contrast, most other thermophilic to extremely hyperthermophilic extrachromosomal elements were in a separate group (cluster c).

The consistency of the 5-mer profile distribution with the “Niche” was lower than that for cells: the “Niche” explained 50.12% of the dissimilarity variance from the extrachromosomal element profiles (Additional File 3, D_5_mobile_ ~ Niche). As we observed for cells, the information about the “Niche” was almost fully included in the host taxonomic classification, since the “Niche” explained only 1.16% of the extrachromosomal element dataset variance when the influence of host taxonomy was first removed (Additional File 3, D_5_mobile_ ~ Host Order*Niche). A statistical model combining the genomic GC content, the ecological niche and the taxonomy of the host explained 70.85% of the profile dissimilarity variance (Additional File 3, D_5_mobile_ ~ Niche*Host Order*GC%); adding the extrachromosomal element family as a variable to the model enabled us to reach 89.29% of explained variance (Additional File 3, D_5_mobile_ ~ Niche*Host Order*GC% and D_5_mobile_ ~ Niche*Host Order*Family*GC%).

### A clear 5-mer signature for halophily and a weaker signature for hyperthermophily

Considering the strong association between the ecological niche and the 5-mer profile distribution, we decided to identify some of the most discriminant 5-mers between halophilic and nonhalophilic entities on the one hand, and between hyperthermophilic versus nonhyperthermophilic entities on the other. For this purpose, in each case, we applied partial least square discriminant analysis (PLS-DA) to archaeal cells and extrachromosomal element profiles separately. In each situation, we retained the ten most discriminant 5-mers (Table 1, Additional file 5).

**Table 1:** Sets of 10 most discriminant 5-mers identified by PLS-DA

	Archaeal cells	Archaeal mobile elements
**Halophiles** **high frequency 5-mers**	**CGAAC**, **GTTCG**, **ACCGA**, GACCG, CGGTC, **TCGGT**, GTGAC, GTCAC, TCGAC	**GTTCG**, **ACCGA**, TTCGA, **CGAAC** TCGAA, **TCGGT**, TCGGA, CGAGT, TCCGA, ATCGA
**Halophiles** **low frequency 5-mers**	TGAAG	–
**Hyperthermophiles** **high frequency 5-mers**	TCAAC, GTTGA, **AGCTT**, **AAGCT**	TTTGG, GAGCT, AGCTC, **AAGCT**, **AGCTT**, TTGAG, (TTGGA), GCCAA, (TCCAA)
**Non-hyperthermophiles** **low frequency 5-mers**	TCAGA, TCTGA, TCAGT, ACTGA, CAGAT, ATCTG	CGAAT

Bold characters: in each table line, most discriminant 5-mers shared between cells and mobile elements, for a considered niche category. In parenthesis: statistically non-significant frequency differences based on a t-test (*p* ≥ 0.01), in a considered niche category

**Fig. 3:**
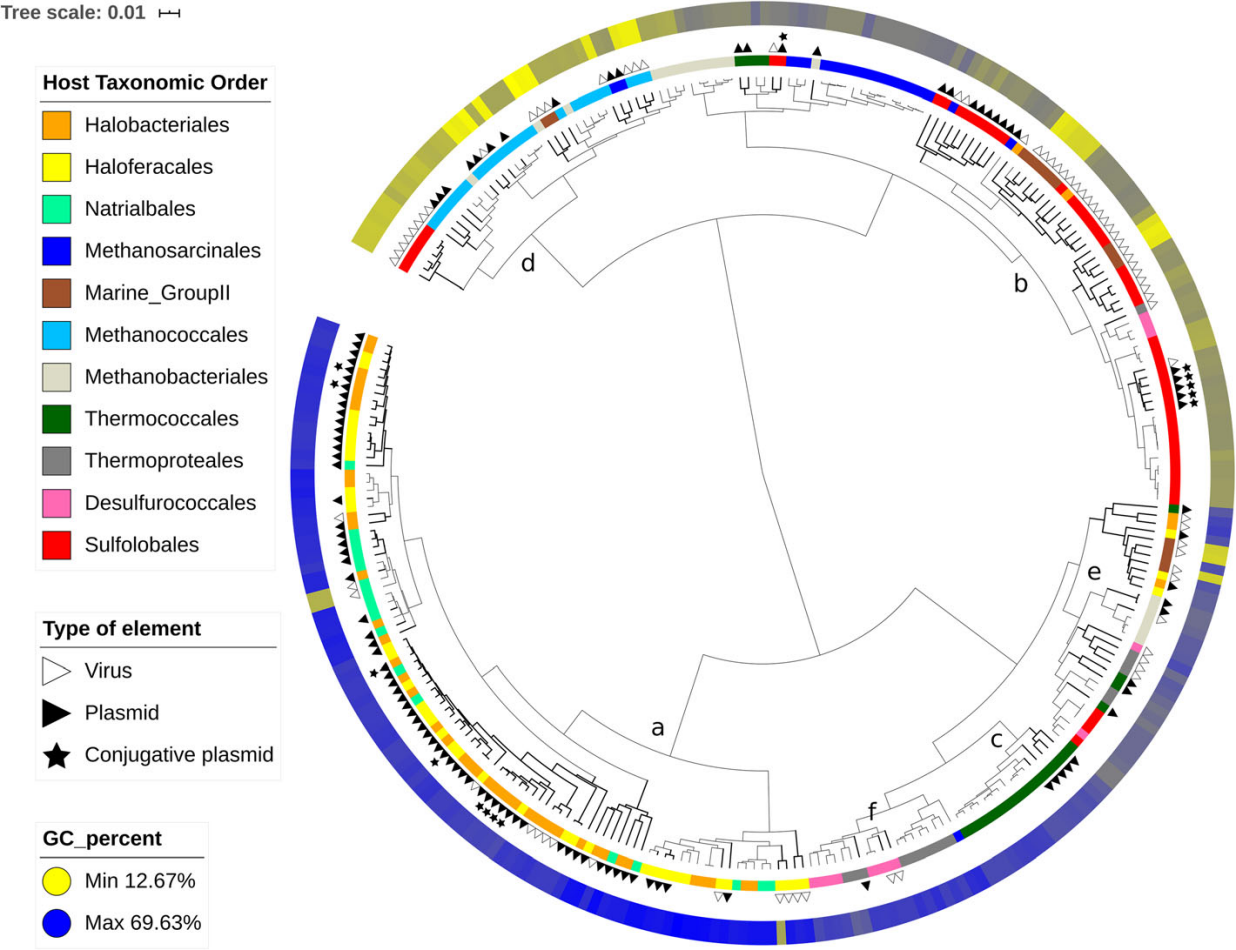
Dendrogram based on 5-mer frequencies for a subset of archaeal cells and mobile elements

For both cells and extrachromosomal elements, the separation according to the salinity-related growth properties was very strong, consistent with the hierarchical clustering results (principal component analysis (PCA) and PLS-DA, Additional files 6, 7, 8, 9). Consistent with this, the average frequency of the ten most discriminant 5-mers was significantly different between halophiles and nonhalophiles (Mann-Whitney-Wilcoxon test, *p* < 0.01, Additional files 10 and 11). Considering the marked separation between halophilic and nonhalophilic entities (Fig. 3, Additional Files 6, 7, 8, 9), many additional 5-mers likely have significantly different frequencies between both groups. The ten most discriminant 5-mers were more abundant in halophilic archaea or in their extrachromosomal elements, except for one 5-mer, which was more abundant in nonhalophilic archaea.

The signatures of halophilic cells and extrachromosomal elements were expected to be similar, since most *Halobacteria* extrachromosomal elements grouped with *Halobacteria* cells in a joint dendrogram (Fig. 3). Indeed, each of the ten discriminant 5-mers identified for the cells also had significantly different frequencies within extrachromosomal elements (Mann-Whitney-Wilcoxon test, *p* < 0.01). However, only 4 out of the 10 most discriminant 5-mers identified for halophiles were common between cells and mobile elements (Table 1, Additional file 5). The 10 most discriminant preferred 5-mers in haloarchaea were GC-rich, as expected (Table 1, Additional file 4).

To identify discriminant 5-mers according to the growth temperature, we removed all *Halobacteria* representatives from the dataset and classified the remaining elements into two categories: elements with growth temperatures below 80 °C (weak mesophiles to extreme thermophiles) and those with growth temperatures above 80 °C (hyperthermophiles to extreme hyperthermophiles).

For archaeal cells, hyperthermophiles and nonhyperthermophiles separated quite well based on PCA and PLS-DA (Additional files 12 and 13). The 10 most discriminant 5-mers identified by PLS-DA all had significantly different frequencies between the two groups (Mann-Whitney-Wilcoxon test, *p* < 0.01, Additional file 14). However, the differences were less pronounced than those for halophiles.

For the extrachromosomal elements, with the same defined categories, the separation between the two temperature groups was less clear, as assessed by PCA (Additional file 15); but the barycenters were still quite distant from each other. Eight of the 10 most discriminant 5-mers identified by PLS-DA (Additional file 16) had significantly different frequencies between the two groups (Mann-Whitney-Wilcoxon test, *p* < 0.01, Additional File 17). Only two of them were shared with those identified for cells, with higher frequencies in hyperthermophiles than in the lower growth temperature group. Seven of the 10 most discriminant 5-mers identified for the cells also had significantly different levels in extrachromosomal elements (Additional file 18), indicating that the signatures of archaeal cells and extrachromosomal elements with respect to hyperthermophily are similar without being strictly identical.

The signal for hyperthermophily was much weaker overall than that for halophily. In addition, most hyperthermophiles in our dataset were from the orders *Desulfurococcales*, *Thermoproteales* and *Thermococcales.* The few others (e.g., some *Sulfolobales* and *Methanococcales* members) tended to be located within the lower-temperature group, as assessed by PCA. It is therefore not clear whether the identified discriminant 5-mers constitute a general signature for hyperthermophilic archaea.

### Codon frequencies influence 3-mer and 5-mer profile distributions

It has been previously shown that amino acid usage and codon frequencies vary according to environmental conditions, particularly for archaea and extreme environments [29, 35, 40, 41]. Since the proportion of coding regions is high in archaeal genomes, it is likely that their 5-mer composition is somehow correlated with the codon frequencies. To evaluate this hypothesis, we focused only on the genomes for which the positions of coding regions were available in public databases, namely 238 out of 239 archaea and 288 out of 345 archaeal viruses and plasmids, in our dataset (Additional file 2).

We first compared, for halophiles and hyperthermophiles, the 10 most discriminant 3-mers of the whole-genome sequences to their 10 most discriminant codons (Table 2). In each case, several of the most discriminant codons were also present among the most discriminant 3-mers of the whole genome sequences (Table 2, underlined words), which supported, as expected, the link between codon frequencies and 3-mer composition in archaea and their extrachromosomal elements.

**Table 2:**
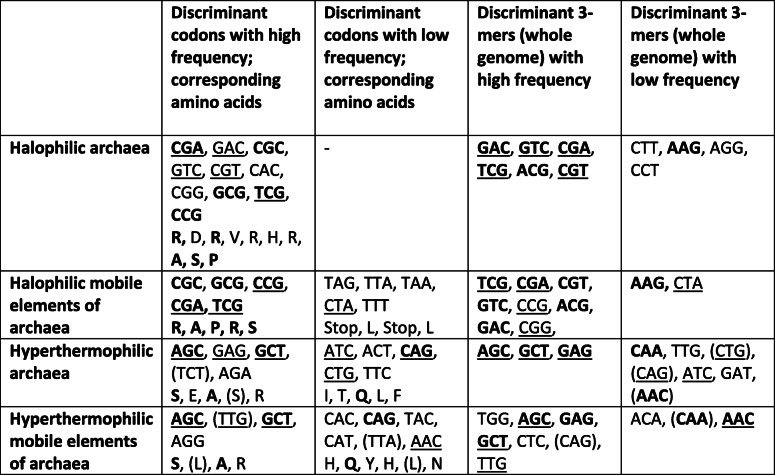
Sets of 10 most discriminant codons and 3-mers identified by PLS-DA

Underlined: most discriminant words shared between codons and 3-mers in whole genomes, for a considered niche category. Bold characters: most discriminant words shared between cells and mobile elements, for a considered niche category. In parenthesis: statistically non-significant frequency differences based on a t-test (*p* ≥ 0.01), in a considered niche category

The 10 most discriminant preferred codons in haloarchaea were GC rich, as expected (Table 2, Additional file 4). They encoded arginine (R) (through 4 different codons), aspartic acid (D), valine (V), histidine (H), alanine (A), serine (S) and proline (P). Contrary to previous results on amino acid composition [35, 41, 42], we did not detect preferred codons for glutamic acid (E) [35, 42, 43] and threonine (T) [35]. D and V have been repeatedly identified as preferred amino acids in halophiles [35, 41, 42]. A higher abundance of R in halophiles has been reported when comparing halophiles to thermophiles [42] or in specific cases [35, 43]; an increase in H has also been documented [41]. The enrichment in R probably compensates for the avoidance of K [35, 41–43]: this latter amino acid is similar to R, a basic, polar and positively charged amino acid; however, the side chains of R can bind more water molecules than those of K. In our study, the identification of 4 preferred codons coding for R could therefore partly result from a selection process operating at the protein level.

Our results on the most discriminant codons for hyperthermophilic archaea can be compared with those from [44], for the identification of differentially abundant codons between thermophilic and mesophilic archaea and bacteria. A limited number of codons identified in [44] were also retrieved in our analysis (Table 2): GAG (E), AGA (R) and AGG (R), which were more frequent in hyperthermophilic archaea or in their extrachromosomal elements; CAG (glutamine, Q), which was less frequent in both hyperthermophilic archaea and their extrachromosomal elements; and finally CAT (H), which was less frequent in hyperthermophilic extrachromosomal elements. However, the majority of the most discriminant codons for hyperthermophily that we identified (Table 2) were not detected as differentially abundant in [44]. In archaea and bacteria, the nature of the discriminant codons is likely influenced by proteomic adaptation to temperature [45]. In 2007, the amino acids isoleucine (I), V, tyrosine (Y), tryptophan (W), R, E and leucine (L) were proposed as universal markers for the optimal growth temperature in prokaryotes (IVYWREL) [45]. These amino acids were already identified to some extent prior to 2007 [44, 46, 47] . Although not present in the IVYWREL set, K was identified by other authors as a preferred amino acid [44, 47]. By contrast, thermophiles tend to be impoverished in at least Q, T and H [44, 46]. Our results on most discriminant codons showed a certain consistency with these established amino acid signatures, since 6 of them translated to one of these amino acids (Table 2, preferred codons translating to E or L and avoided codons translating to Q or H). In our analysis, some codons translating to S, R, and A appeared to be preferred in both hyperthermophilic archaea and their extrachromosomal elements. Finally, 3 avoided codons corresponded to the preferred amino acids I, L, and Y (Table 2), showing the difficulty of fully reconciling the signature at the codon level from this study to the amino acid signature from previous studies.

Examining the influence of codon frequency on the 5-mer profiles is less straightforward, since each 5-mer includes three overlapping 3-mers. We thus implemented a different approach to obtain a global estimate of this influence. We first established another type of 5-mer-based profile, taking into account the codon composition. For each element, this new profile was based on the concatenated coding regions. For each 5-mer, the profile value consisted of an exceptionality score, reflecting how unexpectedly frequent or rare this 5-mer is, considering the codon composition of the sequence. This other type of profile therefore does not necessarily highlight frequent 5-mers. Rather, it highlights 5-mers that have an unexpected frequency in the studied sequence, given the codon frequencies. After obtaining the profiles, we calculated the distance matrices (D_5_cells_e_ and D_5_mobile_e_) before applying PERMANOVA. The influence of the niche was much lower on this new type of profile, decreasing from 64.22 to 41.75% for archaeal cells (D_5_cells_ ~ Niche and D_5_cells_e_ ~ Niche) and from 51.35 to 17.81% for mobile elements (D_5_mobile_ ~ Niche and D_5_mobile_e_ ~ Niche). The strong influence of the ecological niche on the 5-mer profiles is thus significantly but not exclusively explained by codon frequencies.

### Joint analysis of plasmid, viral and cellular genomes from *Archaea* highlights the influence of coevolution and of the extrachromosomal element families on 5-mer profiles

To visualize a dendrogram encompassing both archaeal cells and their extrachromosomal elements, we created a smaller subset by randomly selecting approximately half of the sequences in each category (cell, virus and plasmid) and we jointly analyzed the corresponding 5-mer profiles. This subset comprised a total of 296 genome sequences, of which 119 were from cells, 106 were from plasmids and 71 were from viruses.

Based on hierarchical clustering (Fig. 3) and at the global scale, viruses and plasmids did not form a separate cluster. Rather, they tended to group with archaea sharing the same taxonomy as their hosts. This was best evidenced by the class *Halobacteria*, for which most members and their associated extrachromosomal elements were grouped in a single specific cluster (Fig. 3, letter a). This trend was also visible for the orders *Sulfolobales*, *Thermococcales*, and *Methanococcales* (Fig. 3, clusters b, c, d, respectively). It was less clear for the orders *Methanobacteriales*, *Thermoproteales* and *Desulfurococcales*, as well as *Marine Group II*, which were more dispersed at various locations of the dendrogram. However, several host-virus or host-plasmid associations were still visible in some of these smaller isolated clusters (e.g., for *Methanobacteriales* and *Desulfurococcales*, Fig. 3, letters e and f, respectively). While this trend of 5-mer profile similarity between extrachromosomal elements and hosts has its exceptions, it still highlights the influence of the coevolution between hosts and their mobile elements on their short k-mer composition.

Within each of the 4 abovementioned groups for which the association was the strongest (the class *Halobacteria* and orders *Sulfolobales*, *Thermococcales*, and *Methanococcales*), the cell and extrachromosomal element branches were not fully intertwined. Rather, they tended to form several aggregates rich in either cells or extrachromosomal elements. This is particularly well illustrated by the case of the *Sulfolobales* order (Fig. 3, letter b).

Importantly, although the 5-mer profiles of archaeal extrachromosomal elements are strongly influenced by the coevolution with the hosts, they also retain a specific component, likely due to their different nature. To better understand the nature of these interactions, we focused on *Halobacteria* and *Sulfolobales*, for which many families of extrachromosomal elements, either plasmids or viruses, have already been defined.

### Megaplasmids and other mobile elements from *Halobacteria* have 5-mer profiles distinct from those of *Halobacteria* cells

The class *Halobacteria* comprises exclusively halophilic archaea that thrive in high-salt environments. We focused specifically on the sequenced mobile elements of *Halobacteria* members, which are numerous and diverse [25, 26, 48, 49]. Our dataset comprised 53 cellular *Halobacteria* genomes, as well as 118 plasmids and 36 viruses of hosts from the orders *Halobacteriales*, *Haloferacales*, and *Natrialbales* (Additional file 19). A particularity of *Halobacteria* is the abundance of megaplasmids, considered here as plasmids longer than 150 kb (51 represented in our dataset), and of large plasmids, with sizes ranging from 100 to 150 kb (23 represented in our dataset). The 44 other plasmids had sizes ranging from 1.1 kb to 96 kb. There is currently a scientific debate on the nature of megaplasmids. Indeed, some of them encode essential genes and could hypothetically be currently evolving into chromosomes [50]. In our dataset, 5 distinct elements were classified as second chromosomes according to public databases. Associated with the *Haloarcula* or *Halorubrum* genus, these elements had sizes ranging from 288 kb to 526 kb.

Using PERMANOVA, it appeared again that the genomic GC content and the taxonomic family together explained an important proportion of the 5-mer profile dissimilarity variance of extrachromosomal elements, namely, 55.52% (Additional file 20, D_5_mobile_halo_ ~ GC%*Family). By contrast, the taxonomy of the host explained only a very limited proportion of the variance, 5.28%, consistent with the loss of phylogenetic signal from the hosts within the class *Halobacteria* (Additional file 20, D_5_mobile_halo_ ~ Host order*Host genus).

**Fig. 4:**
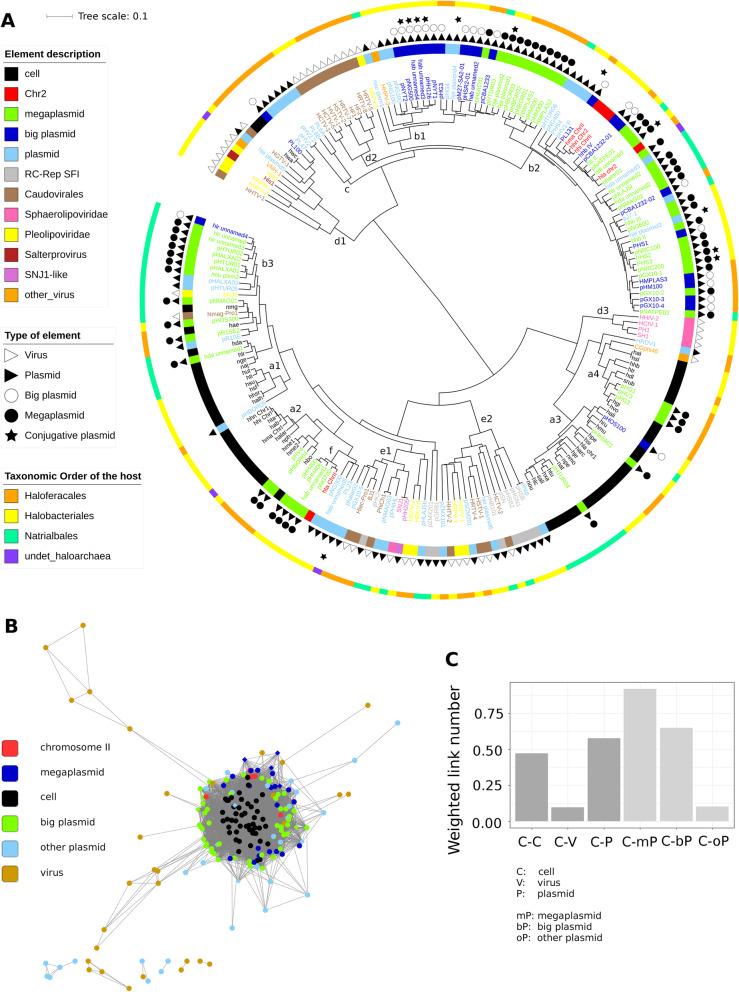
Insight into the archaeal mobile elements from the class *Halobacteria.* **a**. Dendrogram based on 5-mer frequencies for *Halobacteria* members and their plasmids and viruses. **b**. Gene-sharing network based on the normalized number of shared genes. For each pair of elements, the number of shared gene was divided by the lowest genome length of the pair. Moreover, edges with normalized values lower than 0.1 are not shown, to filter out the weak interactions. **c**. Barplot of edge counts from the network according to different categories of elements. The counts were normalized by the number of elements in the considered categories

The pattern obtained by hierarchical clustering was quite complex (Fig. 4a, Additional file 21). It still evidenced the presence of cell-rich clusters (Fig. 4a, clusters a1 to a4), while other clusters were rich in megaplasmids and large plasmids (Fig. 4a, clusters b1 to b3), in other plasmids (Fig. 4a, cluster c), in viruses (Fig. 4a, clusters d1 to d3), or in a mixture of other plasmids and viruses (Fig. 4a, clusters e1 and e2). Some clusters were enriched in plasmids or viruses belonging to well-defined families. In particular, we noticed clusters rich in *Caudovirales* (Fig. 4a, clusters d2), *Sphaerolipoviridae* (Fig. 4a, clusters d3), or RC-Rep SF I elements (Fig. 4a, one subcluster of e2). We also noticed that the *Halobacterium halobium* plasmid ehsp was identical to the *Halobacterium salinarum* plasmid pHSB, a small rolling-circle replication plasmid of 1.7 kb [25] (in cluster e2). For *Caudovirales*, we observed a certain consistency between the viral types and clustering patterns. Except for HHTV-1, HGTV-1 and the *Natrialba magadii* provirus (Nmag-Pro1), *Caudovirales* members were distributed among 3 main clusters (Fig. 4a, cluster d2, one subcluster of e1, one subcluster of e2). The first one exclusively comprised 9 *Caudovirales* members (Fig. 4a, cluster d2), with an average genome length of 83.3 kb. Within this cluster, the 3 HCTV-type *Siphoviridae* members grouped together (HCTV-1, HCTV-5 and HVTV-1); in the *Myoviridae* family, similar results were observed for the 4 HF2-type viruses (HF1, HF2, HRTV-8 and HRTV-5) and for both HRTV-7-type viruses (HRTV-7 and HSTV-2). Moreover, HF2-type and HRTV-7-type viruses that are evolutionarily related [49] also clustered together. In contrast, other *Caudovirales*-rich clusters also comprised plasmids of limited size as well as *Pleiolipoviridae* and *Sphaerolipoviridae* members. *Caudovirales* members in these mixed clusters had a smaller average genome size, of 43.5 kb. Finally, HHTV-1 (*Caudovirales* order) was one of the outermost elements in the haloarchaea dendrogram (Fig. 4a, in cluster d1), consistent with its description as the most divergent among sequenced haloarchaeal tailed viruses [49].

A gene-sharing network based on protein similarity was constructed (Fig. 4b) and supported the same observation when the weak edges were filtered out. This reinforces the conclusion since gene sharing networks address a different type of information, depending on the genome functional content.

The network (Fig. 4b) also showed that cells shared few strong edges with plasmids of limited size (< 100 kb), in contrast to large plasmids and megaplasmids. This was further confirmed by basic statistics on the number of edges among these different types of elements (Fig. 4c). For the smaller plasmid category (< 100 kb), the level of this indicator was actually similar to that of viruses (Fig. 4c). *Halobacteria* plasmids therefore seem to have heterogeneous properties with respect to genetic connections with their hosts. Plasmid size appears to act as a major influential factor, possibly by increasing the probability of gene exchange.

### Good congruence between mobile element families and 5-mer composition in *Sulfolobales*

Viruses and plasmids present in *Sulfolobales* (genera *Sulfolobus*, *Metallosphaera* and *Acidianus*) are among the best characterized archaeal mobile elements. *Sulfolobales* members produce viruses with unique morphotypes (e.g., fusiform, bottle-shaped), which has aroused important scientific interest during the last two decades [51]. *Fuselloviridae*, *Lipothrixviridae*, and *Rudiviridae*, reviewed in [24]) and 2 distinct plasmid families (cryptic pRN-like, conjugative pNOB8-like [52]) have been studied extensively. A total of 119 *Sulfolobales* sequences of cells, plasmids and viruses were studied here (Additional File 22).

**Fig. 5:**
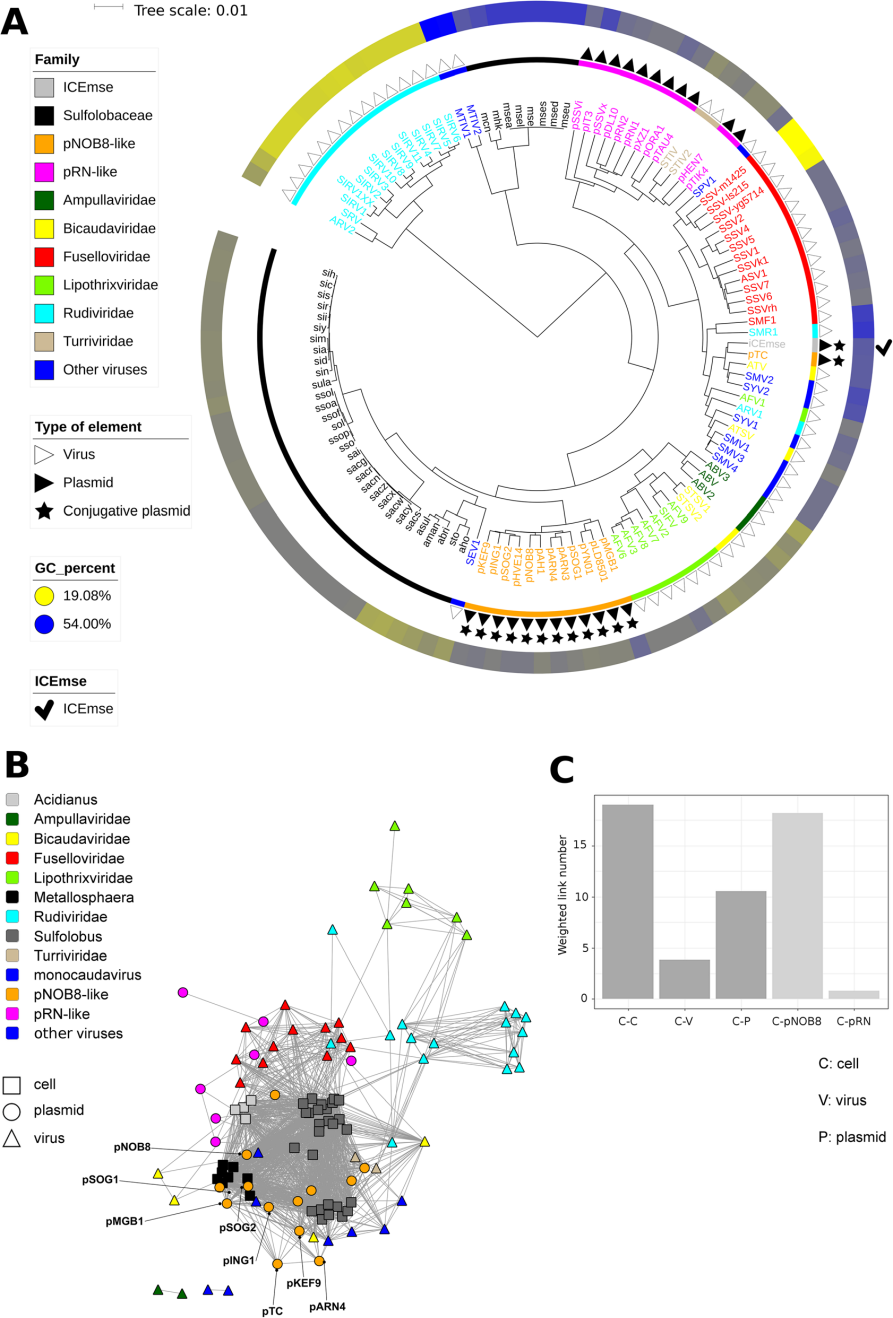
Insight into the archaeal mobile elements from the order *Sulfolobales.* **a**. Dendrogram based on 5-mer frequencies for *Sulfolobales* members and their plasmids and viruses. **b**. Gene-sharing network based on the normalized number of shared genes. For each pair of elements, the number of shared gene was divided by the lowest genome length of the pair. Moreover, edges with normalized values lower than 0.1 are not shown, to filter out the weak interactions. **c**. Barplot of edge counts from the network according to different categories of elements. The counts were normalized by the number of elements in the considered categories

The cellular genomes were distributed between 2 distant clusters, one corresponding to *Metallosphaera* and the other to *Sulfolobus* and *Acidianus* (Fig. 5a, black color, codes starting with m, s and a respectively). The average genomic GC content in *Metallosphaera* was of 45.4% ± 1.6 SD, compared to 35.2% ± 1.6 SD in the other *Sulfolobales* genomes, which possibly influenced this partition. In the *Sulfolobus-Acidianus* cluster (Fig. 5a), the subclusters were consistent with the distinct species, namely, *Sulfolobus islandicus* (codes starting with si), *Sulfolobus solfataricus* (codes starting with sso or so), *Sulfolobus acidocaldarius* (codes starting with sac or sa) and *Acidianus* species (codes starting with a). The only exception was *Sulfolobus tokkodai* (code sto), which was located in the *Acidianus* subcluster.

The *Sulfolobales* extrachromosomal elements were grouped primarily according to their taxonomic family rather than to the taxonomy of their hosts (Fig. 5a). This general pattern appeared once more to be partly linked to the GC content of the sequences (Fig. 5a, Additional file 23). There were notable exceptions, such as the *Fuselloviridae* proviruses previously described in [53] (Fig. 5a, SSV-m1425, SSV-ls215 and SSV-yg5714): their sequences were less GC rich than those of the other *Fuselloviridae* members (19.6% ± 0.7 SD compared to 39.2% ± 2.3 SD) but they were still located in the main *Fuselloviridae* cluster.

For viruses, 14 out of 16 *Rudiviridae* genomes, 12 out of 13 *Fuselloviridae* genomes and 7 out of 8 *Lipothrixviridae* genomes clustered together (Fig. 5a). A similar trend was observed for less represented families, with all *Ampullaviridae* and *Turriviridae* members grouping into consistent clusters. For the plasmids, all pRN-like cryptic plasmids and 2 related phage-plasmid hybrid entities (pSSVx and pSSVi) (Fig. 5a, magenta color) formed a single cluster that also included *Turriviridae*. Finally, 12 out of the 13 pNOB8-like conjugative plasmids clustered together (Fig. 5a, green color). Interestingly, the main pNOB8-like plasmid cluster (with sizes ranging from 20.4 to 42.2 kb) was located very close to the main cell cluster, whereas the pRN-like cryptic plasmid cluster (with sizes ranging from 5 to 13.6 kb) was much more distant (Fig. 5A). Similar to our observations for *Halobacteria*, this finding highlights that larger plasmids are more similar to cells than shorter plasmids and viruses in terms of 5-mer composition.

This could reflect the occurrence of frequent genetic exchange between *Sulfolobales* cells and pNOB8-like conjugative plasmids. Based on PERMANOVA, the viral and plasmid families together with the genomic GC content explained 77.68% of the 5-mer profile dissimilarity variance among *Sulfolobales* mobile elements (Additional file 23, D_5_mobile_sulfo_ ~ Family*GC%).

A gene sharing network also showed that *Sulfolobales* mobile elements tended to group according to their family. The proximity of pNOB8-like conjugative plasmids and *Sulfolobales* cells was visible, whereas connections between cells and pRN-like plasmids or viruses were less striking (Fig. 5b, Fig. 5c). A noticeable difference between the dendrogram based on the 5-mer profiles and the gene sharing network regarded the links between the *Lipothrixviridae* and *Rudiviridae* families, which together form the *Ligamenvirales* order [54]. While this evolutionary connection was clear in the gene sharing network (Fig. 5b), it was not clear from the 5-mer-based analysis (Fig. 5a), confirming the idea that sequence composition changes more rapidly than gene content and that similarity in sequence composition can identify only close evolutionary relationships. The different 5-mer compositions between *Lipothrixviridae* and *Rudiviridae* may be explained by the low genomic GC contents of *Rudiviridae* (28.25% ± 6.17% SD on average). We also noticed that *Rudiviridae* members seem to have an unusual 5-mer composition since their main cluster had a long branch and they were isolated not only from *Lipothrixviridae* but also from all other mobile elements (Fig. 5a). In addition to their very low GC content, several factors could possibly explain the specific 5-mer composition of *Rudiviridae*, such as unusual DNA packaging constraints or their DNA replication mode (hypothetically complex mechanisms, not yet fully identified [55], reviewed in [24]).

### Outliers and host transfers

Genomes with unexpected 5-mer composition (outliers) could presumably reveal singular evolutionary trajectories. We identified a total of 51 outlier plasmids and viruses (Additional File 2) by combining a systematic approach (see Materials and Methods) and visual examination of the dendrograms. These elements had unexpected 5-mer compositions compared to the average in their taxonomic group or the 5-mer composition of their hosts.

For 4 of them, their very short length (< 4 kb) likely explains their atypical composition. The presence of tRNA genes in viral genomes has previously been identified as a possible factor explaining the divergence between host and viral genome k-mer compositions, acting by reducing the selective pressure on the viral genome for adaptation to host codon usage [14, 56]. Such a phenomenon was not prevalent here, since only 3 out of 51 outliers encoded tRNAs in their genomes (Additional File 2).

**Fig. 6:**
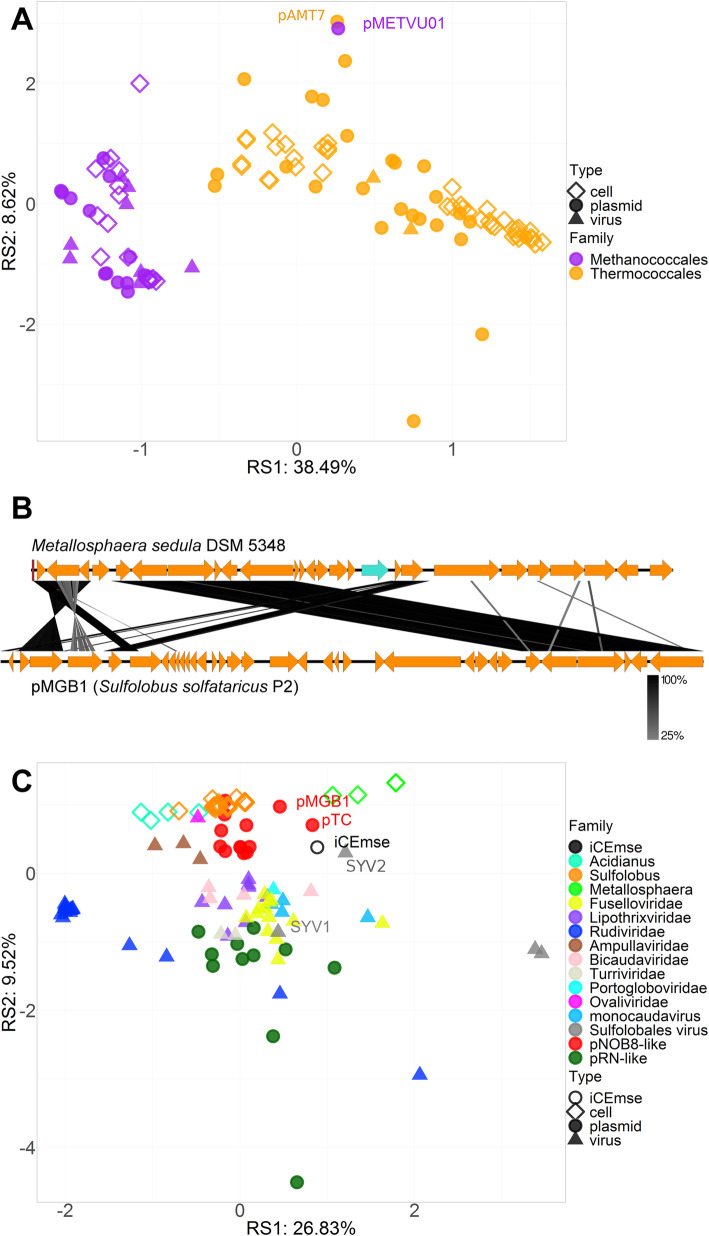
llustration of host transfer events. **a**. PCA highlighting the recent interorder transfer of a *Methanococcales* plasmid from the *Thermococcales* order. **b**. Comparison of pMGB1, a *Sulfolobus* plasmid of the pNOB8-like conjugative family, with a selected region of *Metallosphaera sedulla* DSM 5348 genome, showing the intergenus transfer. **c**. PCA of *Sulfolobales* cells, viruses and plasmids, as well as the newly identified Integrative Conjugative Element present in *Metallosphaera sedulla* DSM 5348 genome (iCEmse)

Assuming that recent host transfer could also explain atypical 5-mer compositions, we specifically examined *Thermococcales* and *Methanococcales*, which are evolutionarily closely related and known to share evolutionarily-related plasmids. One of the previously described interorder host transfer events was indeed visible by PCA (Fig. 6a) or hierarchical clustering (Additional File 24), suggesting that the *Methanocaldococcus* plasmid pMETVU01 originated from a *Thermococcales* host [25]. More ancient evolutionary connections detected previously between some *Methanococcales* plasmids, such as pMEFER01, and the pT26–2 *Thermococcales* plasmid family [25] were not visible based on the 5-mer profiles. This suggests that the 5-mer composition of newly transferred mobile elements must evolve rapidly, so only recent transfers can be detected by this approach.

We then considered more closely the 13 pNOB8-like *Sulfolobales* conjugative plasmids because in a previous version of the dataset, two pNOB8-like plasmids, namely, pMGB1 and pTC, were located close to *Metallosphaera* genomes, far from the main pNOB8-like cluster (Additional File 25). This suggested that pTC and pMGB1 could replicate in *Metallosphaera* archaea, in addition to *Sulfolobus*. Interestingly, we identified a remnant plasmid very similar to pMGB1 in the genome of *Metallosphaera sedula* (Fig. 6b), consistent with this hypothesis. We named this new integrated conjugative plasmid ICEmse, for “Integrative Conjugative Element of *M. sedula*”, and we included it in the dataset. ICEmse was consistently located in the same cluster as the pTC, pMGB1 and *Metallosphaera* genomes in the previous dataset version (Additional File 26). In our latest dataset version, the trends were less clear, since *Metallosphaera* formed a fully separate cluster. Moreover, only pTC grouped with ICEmse (Fig. 5a) and was detected as an outlier. By contrast, pMGB1 was located in the main pNOB8-like plasmid cluster, but was the outermost element. The PCA result was in good agreement with the host transfer scenario, since pTC, pMGB1 and ICEmse were located roughly at mid-distance between *Sulfolobus* and *Metallosphaera* cells (Fig. 6c). Finally, consistent with the high GC content of *Metallosphaera* genomes, the pMGB1, pTC and ICEmse genomic GC contents were 39.6, 41.4 and 41.5%, respectively, compared to only 36.7% ± 0.6 SD for the other pNOB8-like elements, again supporting the host transfer hypothesis.

## Discussion

The influence of their phylogenetic position on the 5-mer composition of archaeal cell genomes is clearly visible in our dataset, consistent with the genome-wide importance of short k-mers, which could play a role in speciation and be critical to recombination (reviewed and defended in [2]). However, the global topology that we obtained by hierarchical clustering was not fully consistent with the phylogeny of archaea, as detailed in the results section. It could be interesting to evaluate whether more sophisticated methods [16–18] and the use of various k-mer sizes would enable us to obtain a global topology more consistent with the phylogeny of archaea. Whether it could be achieved is, however, uncertain. The fact that we could detect recent HGTs but that several ancient evolutionary connections [54, 57] were not detected in our analysis suggests that the genome composition in short k-mers must evolve rapidly. The acquisition or loss of adaptation to extreme conditions played a strong role in the evolution of archaea (e.g. [29, 34]). It was proposed that the last archaeal common ancestor was a hyperthermophile [29], and the subsequent adaptation to other niche constraints may likely have blurred the phylogenetic signal of k-mer profiles in *Archaea*. This must have resulted in certain cases in convergent evolution of sequence composition, which could also blur the phylogenetic signal.

Our results were mostly consistent with previous studies, but they provide a different view since most of the latter focused on amino acid composition [35, 41, 42] and codon usage (e.g., [35]), rather than k-mers and absolute codon frequencies. Our analysis shows that the ecological niche also has a strong link with the 5-mer composition of archaeal extrachromosomal elements. For virions in particular, it would be interesting to determine whether the composition results exclusively from the coevolution with the hosts or whether other selective pressures are exerted, for instance on the packaging structure properties during the extracellular stage, corresponding to a more direct effect of the extracellular environment.

*Halobacteria* members and their extrachromosomal elements showed a very strong signature at all studied levels: GC content, 5-mer and 3-mer compositions of the whole genome sequences and codon composition. *Halobacteria* was clearly separated from the other clades of archaea, most likely as a consequence of their evolution in high-salt environments. Halophiles have an exceptionally high GC content among archaea (~ 60%) (Additional file 4), possibly to prevent the formation of thymidine dimers following extensive exposure of these archaea to UV at the surface of solar salterns [58]. *H. walsbyi* genomes are notable exceptions, and their low GC-content (48%) may be partly compensated by the presence of 4 encoded photolyases in their genomes [59]. In addition, proteins of halophiles have specific features that enable them to be functional under the high salt concentration in the cytoplasm (up to 4 M KCl) [35]. Their surface is typically enriched in acidic [42] and negatively charged residues [43], while their core has a moderate hydrophobicity [43].

Regarding the signature for hyperthermophily, many differences in the methods and datasets could explain the imperfect agreement with previous studies [44, 45]. Primarily, our information on amino acids is indirect, based on absolute codon frequency analysis, while most cited studies directly focused on amino acid composition. An additional explanation could be that several previous analyses included both archaea and bacteria, whereas we focused exclusively on archaea, mainly on *Desulfurococcales*, *Thermoproteales* and *Thermococcales*. In addition, our dataset includes more sequences, and finally, the statistical methods employed are slightly different. In particular, Lambros et al. [60] considered the optimal growth temperature as a quantitative variable, pointing out that most changes in response to growth temperature occur below 60 °C. We therefore may have missed some of the compositional changes that start to occur at lower temperatures. It is, however, interesting that discriminant 5-mers could be identified from our diverse dataset and when considering a high temperature threshold to partition the dataset into two categories.

We observed that mobile elements of archaea harbor some specificity in their 5-mer composition compared to their hosts, with two major types of situations. The first corresponds to major compositional differences between the mobile elements and their hosts. Such mobile elements are outliers and do not represent the most frequent cases. According to the literature, such differences could be explained by the presence of tRNA genes in the mobile element genome, enabling the uncoupling of codon usage constraints of the hosts from those of the mobile element [14, 48]; by a large genome size of the mobile element, which is indicative of a more autonomous replication cycle [14]; or by a recent acquisition by the host, such that the composition of the mobile element has not yet undergone host adaptation [31]. In the present study, we found a very limited presence of annotated tRNA genes in mobile elements (Additional file 2). We identified two recent host transfers, one previously described (pMETVU01) [25] and a newly described one (ICEmse). We hypothesize that the fact that the *Halobacteria* viruses His1 and His2 encode their own family B DNA polymerase [24] could possibly contribute to their atypical 5-mer composition. Apart from these few cases, no obvious factors could be identified at first glance for most outliers.

A second type of case, the most frequent, corresponds to a small 5-mer composition difference between the mobile elements and their hosts. In the literature, the influence of the host range and mode of transmission have been proposed, such as frequent changes of hosts [31] or a wide host range [19]. For horizontally transferred mobile elements, occasional exposure to the extracellular environment could also create particular selective pressures [31]. Competition for metabolic resources has also been suggested to explain differences in GC content [61]. Beyond these general factors, we suggest that the specific composition of mobile elements could primarily result from the intrinsic properties of mobile element families. This idea is best illustrated by *Sulfolobales* plasmids and viruses that cluster mainly according to their own taxonomic family, rather than those of their host strains. This suggests that each mobile element family has its own specificity in terms of 5-mer composition and indicates that their 5-mer composition does not simply reflect their adaptation to their hosts or to the extracellular environment. This notion is echoed by [15], the authors of which could classify viruses based on their tetramer composition. One could imagine other selective forces shaping the k-mer composition of mobile elements. There could hypothetically be constraints related to the replication mode or the functional content. For plasmidions [62, 63] and viruses, additional constraints linked to packaging or structure can be imagined, in relation to but not limited to the properties of the extracellular environment.

Interestingly, we observed a lower difference in sequence composition between hosts and large plasmids or megaplasmids, than between hosts and smaller plasmids and viruses. A similar trend was previously observed by several authors who suggested that the low difference in the case of large plasmids could be explained by a stronger adaptation to the host for large plasmids [32] whereas the larger difference in the case of small plasmids could result either from the limited compositional representativeness of short sequences [32] or by their greater host range [19]. We hypothesize that the lower difference in the case of large plasmids could also be due to the fact that they exchange more genes with their hosts and also lack the selective pressures related to packaging or stability in the extracellular environment. Paul et al. [35] mentioned that the difference in codon usage between chromosomes I and II of *Haloarcula marismortui* must be linked to the more recent acquisition of the second chromosome. Our study shows that second chromosomes in the class *Halobacteria* have a 5-mer signature similar to that of large or megaplasmids, and distinct from that of first chromosomes. Therefore, the distinct nucleotide composition of chromosome II of *H. marismortui* could also result from its different origin from that of chromosome I, supporting the idea that chromosome II belongs to the plasmid realm.

Our simple gene sharing network analyses yielded consistent trends, again highlighting a stronger link between larger plasmids and cells than between short mobile elements (plasmids or viruses) and cells. Similar analyses have previously highlighted the important role of mobile elements in gene dissemination, enabling the identification of those more specifically involved in this process [64, 65]. Halary et al. [65] in particular contrasted viruses and plasmids, the latter being, according to their study, the major key players of HGT. Even if our study covers a single domain of life, our observations suggest that the size of the mobile elements (plasmid or viruses) might be in fact the most important factor determining its importance in the evolutionary relationships with hosts. Moreover, the delineation between plasmids, viruses and other types of mobile elements, such as plasmidions, is becoming increasingly blurred [62].

## Conclusions

Our study provides a useful framework for the interpretation of k-mer approaches applied to cell or extrachromosomal elements of the domain *Archaea*. For cells, the global topologies based either on 5-mer profiles or on phylogeny are inconsistent. At a finer level, the results, however, show the strong influence of phylogenetic relationships and of adaptation to environmental constraints on 5-mer compositions. These two factors are interdependent to a significant extent, and the respective weight of their contribution varies according to the clade. Our analysis highlighted the possibility of differential adaptation to the environmental niche between chromosomal DNA and extrachromosomal element DNA. In addition, we clearly observed different patterns depending on the mobile element type and size. For mobile elements, coevolution with the host has a clear influence on their 5-mer composition. However, strikingly, viral and plasmid families also retain a specific imprint in their 5-mer profile. Our analysis also enabled us to detect two host transfer events, but exclusively recent ones, which suggests the fast adaptation of short k-mer profiles in a fluctuating environment. The genome composition difference observed here between mobile genetic elements and their hosts suggests that using k-mer based methods to analyze mobile elements in metagenomic data may lead to spurious results. Incorrect host prediction could occur [66], as well as missed detection of integrated elements during MAG reconstruction [67].

Our results thus call for caution when using k-mers for the identification of mobile elements in metagenomics data, for host prediction of mobile elements, and for phylogenetic reconstruction, especially for ancestral events.

## Methods

### Presentation of the dataset and of the approach

Basic information about the genomes included in the dataset is available in Additional file 2, such as the taxonomy, length and GC content of each element. Additional file 4 provides a synthetic view of GC% values across the dataset, according to the taxonomic order of the host and to the type of element; Additional file 27 shows the GC% values according to the Niche and type of element; finally, an analysis of variance (ANOVA) of these GC% values is presented in Additional file 28.

We selected 11 taxonomic groups (at the order level) of the domain *Archaea* (Additional files 19 and 29) for which a significant number of extrachromosomal element sequences were available (plasmids or viruses). For these 11 taxonomic orders, we gathered a total of 589 whole genome sequences of cells, plasmids, viruses and proviruses. The dataset covered 3 and 8 orders of the phyla *Crenarchaeota* and *Euryarchaeota*, respectively. It comprised exclusively halophiles, acidothermophiles, hyperthermophiles and methanogens.

For each genome, we established a profile consisting of its 5-mer absolute frequencies. To select the k-mer length, a compromise needed to be established: longer k-mers are more informative; however, excessively long k-mers result in data scarcity due to low average counts, leading to artifacts during subsequent statistical analyses. For plasmids and viruses, k-mer length of 5 was selected as a good compromise. Indeed, their average genome length in the dataset was 89,814 bases; since there are 4^k^ distinct possible k-mers, the average counts were 88 per 5-mer (89,814 divided by 4^5^), which we considered sufficiently representative, and slightly more specific than tetramers. For cells, although they have a much higher average genome length, we also used 5-mers to compare their profiles with those of extrachromosomal elements.

The obtained 5-mer frequency profiles included 1024 proportions (4^5^) and constituted a highly multidimensional dataset. To gain insight into these complex data, the landscape of these profiles across the dataset was explored with four methods: hierarchical clustering, PCA, PERMANOVA and PLS-DA. PCA aims to project highly multidimensional data on a set of orthogonal axes to visualise them easily while preserving their variance as best possible. PERMANOVA is a generalized form of ANOVA used to analyze the variance of multidimensional values, here the 5-mer profile distance matrix, and relate them to potential structuring factors. Finally, PLS-DA was used to identify the most discriminant k-mers between several categories of genomes, such as genomes from halophiles, versus nonhalophiles.

### Genome sequences

We collected 534 publicly available whole genome sequences of cells, plasmids, viruses and proviruses (Additional file 2) from the NCBI genome database. We performed a final update on the 7th of August 2018. In addition, we retrieved 28 provirus sequences directly from cellular genome sequences based on literature information [53, 68, 69]. Finally, we included 26 magrovirus sequences [70] available on a specific website (https://github.com/BejaLab/Magrovirus/tree/master/Supp_files) and the assembly of a Marine Group II archaeon (GCA_003324605). When the mobile elements were not classified into well-defined families, we categorized them according to the taxonomy of their host (e.g. *Halobacteriales* megaplasmid).

### Establishment of profiles based on the sequence 5-mer composition

Two types of profiles were established for each sequence based on its 5-mer composition, as described in more detail below. The profiles of the different genomes were then combined across the dataset to obtain two distinct matrices, one for each type of profile.

The first type of profile was based on the 5-mer frequencies of the whole genome sequences. The 5-mer counts were calculated with Jellyfish 2.2.6 on the INRAE-MIGALE cluster (URL https://migale.inrae.fr/). The obtained count data were imported into R [71] (version 3.4.2) and transformed into a frequency matrix to obtain normalized data: for each genome, the sum of the 5-mer frequencies was equal to 1.

The second type of profile relied exclusively on the coding regions; it reflected the exceptionality of the different 5-mers in the coding regions after correcting for differences in codon composition in the studied genome. The exceptionality scores were calculated with R’MES software [72], with the following options: Gaussian model, k-mer length of 5, second-order Markov chain model, and 3 phases. Briefly, R’MES fits a Markov chain on each genome’s concatenated coding regions to compute the expected frequencies of 5-mers based on observed codon frequencies. Exceptionality scores are then computed as standardized deviations between observed and expected 5-mer frequencies. The exceptionality score values obtained for each 5-mer were directly used to generate the second type of 5-mer profile of each genome. R’MES was run on the INRAE-MIGALE cluster.

### Statistical analyses of the profiles based on 5-mer composition

All statistical analyses were performed using R (version 3.4.2). PCA were performed with the dudi.pca function of the ade4 package [73], on scaled and centered data. We performed PLS-DA analyses with the caret package [74], using a 10-times repeated 10-fold cross-validation and the “accuracy” metrics to select the number of components, again on centered and scaled data. Hierarchical clustering was realized with the hclust function from R applied to Euclidian distance matrices with the Ward.D2 method. PERMANOVA of Euclidian distance matrices were conducted with the adonis function of the vegan package [75], with *p*-values computed on 9999 permutations. PERMANOVA assumes that 5-mer profiles respond linearly to changes in the covariates and that the variance of profiles is comparable across conditions of the data. The *p*-values were computed by permutations: this nonparametric approach is robust to model misspecification. The wilcox.test function from R CRAN was employed to test the equality of means through Mann-Whitney-Wilcoxon statistical tests.

Most plots were prepared with ggplot2 package [76]. Dendrograms constructed with hclust were exported in newick format and used in the online tool Interactive Tree Of Life (iTOL) [77] to construct the tree figures.

### Network analyses

Gene sharing network data were generated with EGN 1.0 software [78]. For this purpose, whole proteomes were downloaded from the NCBI website; the resulting multifasta file was formatted according to the EGN manual’s instructions. Blastp [79] searches were computed within EGN software, which acts as a wrapper. The EGN parameters were set as follows: e-value threshold of 1e-05, hit identity threshold of 30%, hit coverage of the shortest sequence of 60%, hit coverage of both sequences of at least 30%, minimal hit length of 20 amino acids, best reciprocity threshold of 10%. The EGN results consisted in the number of similar genes shared between each pair of genomes. These values were subsequently normalized by dividing them by the smallest genome length of the concerned pair.

The obtained networks were visualized with Cytoscape 3.7.1 [80] by using the edge-weighted spring embedded layout and by filtering out the weaker interactions (edge values), as specifically indicated in each case.

### Genome comparison

BLAST comparisons between selected genomes were visualized with Easyfig 2.2.2 [81].

### Outlier identification

For each viral or plasmid family, the distance of each element’s 5-mer profile to the profile barycenter of the considered family was calculated. A gamma distribution was fitted to the histogram of all distances. A 0.95 confidence threshold was selected to define outliers, corresponding to a distance value of 1.654. With this approach, implemented by a homemade R script, 18 outliers were identified, of which 3 were removed after visual examination of the 5-mer frequency-based dendrograms. In addition to this systematic method, 36 other outliers were identified by visual examination of these dendrograms (e.g. genomes not clustering with other genomes from the same family), resulting in a total of 51 outlier elements.

## Supplementary Information

**Additional files 1 and 3 to 29.** Additional tables, figures and text.

**Additional file 2.** Excel file with genome list and genomic features.

## Data Availability

“The dataset supporting the conclusions of this article is included within the article and its additional files.”

## Abbreviations

ANOVA: Analysis of variance
HGT: Horizontal gene tranfer
MAG: Metagenome-assembled genome
PCA: Principal component analysis
PERMANOVA: Permutational multivariate analysis of variance
PLS-DA: Partial least squares discriminant analysis
A: Alanine
D: Aspartic acid
E: Glutamic acid
F: Phenylalanine
H: Histidine
I: Isoleucine
L: Leucine
N: Asparagine
P: Proline
Q: Glutamine
R: Arginine
S: Serine
T: Threonine
V: Valine
W: Tryptophane
Y: Tyrosine
