# Supplementary material for: Exploring short k-mer profiles in cells and mobile elements from Archaea highlights the major influence of both the ecological niche and evolutionary history
Source: BMC Genomics. 2021 Mar 16;22:186. doi: 10.1186/s12864-021-07471-y (PMC7962313; doi:10.1186/s12864-021-07471-y)
Supplement: Supplementary file 1 — Additional files 1 and 3 to 29. Additional tables, figures and text. [file 12864_2021_7471_MOESM1_ESM.pdf]

Additional File 01. PERMANOVA analyses of the 5-mer profiles from archaeal cells

N°	Model	Factor	Explained variance	Statistical significance
1	D_{5_cells}~GC%	GC%	69.10%	1e-04 ***
2	D_{5_cells} ~Phylum*Order*Genus	Phylum	7.06%	1e-04 ***
		Order	68.89%	1e-04 ***
		Genus	17.74%	1e-04 ***
3	D_{5_cells} ~Order	Order	75.94%	1e-04 ***
4	D_{5_cells} ~Niche	Niche	64.17%	1e-04 ***
5	D_{5_cells} ~Order*GC%	Order	75.94%	1e-04 ***
		GC%	12.39%	1e-04 ***
		Order : GC%	4.95%	1e-04 ***
6	D_{5_cells} ~GC%*Order	GC%	69.10%	1e-04 ***
		Order	19.23%	1e-04 ***
		GC% : Order	4.95%	1e-04 ***
7	D_{5_cells} ~Niche*Order	Niche	64.17%	1e-04 ***
		Order	15.38%	1e-04 ***
		Niche : Order	3.22%	1e-04 ***
	D_{5_cells} ~Order*Niche	Order	75.94%	1e-04 ***
		Niche	3.60%	1e-04 ***
		Niche : Order	3.22%	1e-04 ***
9	D_{5_cells} ~Niche*GC%	Niche	64.17%	1e-04 ***
		GC%	19.91%	1e-04 ***
		Niche : GC%	3.72%	1e-04 ***
10	D_{5_cells} ~Niche*Order*GC%	Niche	64.17%	1e-04 ***
		Order	15.38%	1e-04 ***
		GC%	10.30%	1e-04 ***
		Total interaction terms	5.63%	
		Residuals	4.52%	

Additional file 02: Excel file with genome list and genomic features

Additional File 03. PERMANOVA analyses of the 5-mer profiles from archaeal viruses and plasmids

N°	Model	Factor	Explained variance	Statistical significance
1	D_{5_mobile}~ Type (virus / plasmid)	Type	6.14%	1e-04 ***
2	D_{5_mobile} ~Genome length	Genome length	10.25%	1e-04 ***
3	D_{5_mobile} ~GC%	GC%	45.13%	1e-04 ***
4	D_{5_mobile} ~Niche	Niche	50.12%	1e-04 ***
5	D_{5_mobile} ~ Host Phylum* Host Order* Host Genus	Host Phylum	16.59%	1e-04 ***
		Host Order	40.77%	1e-04 ***
		Host Genus	8.03%	1e-04 ***
6	D_{5_mobile} ~Host Order	Host Order	57.36%	1e-04 ***
7	D_{5_mobile} ~Family	Family	68.30%	1e-04 ***
8	D_{5_mobile} ~Niche*GC%	Niche	50.12%	1e-04 ***
		GC%	6.64%	1e-04 ***
		Niche : GC%	4.19%	1e-04 ***
9	D_{5_mobile} ~GC%*Niche	GC%	45.13%	1e-04 ***
		Niche	11.63%	1e-04 ***
		GC% : Niche	4.19%	1e-04 ***
10	D_{5_mobile} ~Host Order*GC%	Host Order	57.36%	1e-04 ***
		GC%	5.42%	1e-04 ***
		Host Order : GC%	5.68%	1e-04 ***
11	D_{5_mobile} ~GC%*Host Order	GC%	45.13%	1e-04 ***
		Host Order	17.65%	1e-04 ***
		GC% : Host Order	5.68%	1e-04 ***
12	D_{5_mobile} ~Family*GC%	Family	68.30%	1e-04 ***
		GC%	6.25%	1e-04 ***
		Family : GC%	8.72%	1e-04 ***
13	D_{5_mobile} ~GC%*Family	GC%	45.13%	1e-04 ***
		Family	29.42%	1e-04 ***
		GC% : Family	8.72%	1e-04 ***
14	D_{5_mobile} ~Host Order * Family	Host Order	57.36%	1e-04 ***
		Family	16.40%	1e-04 ***
		Host Order : Family	2.56%	1e-04 ***
15	D_{5_mobile} ~Family*Host Order	Family	68.30%	1e-04 ***
		Host Order	5.46%	1e-04 ***
		Family : Host Order	2.56%	1e-04 ***
16	D_{5_mobile} ~Family*Niche	Family	68.30%	1e-04 ***
		Niche	4.00%	1e-04 ***
		Family : Niche	3.08%	1e-04 ***
17	D_{5_mobile} ~Niche*Family	Niche	50.12%	1e-04 ***
		Family	22.18%	1e-04 ***
		Niche : Family	3.08%	1e-04 ***
18		Host Order	57.36%	1e-04 ***

	D_{5_mobile} ~Host Order * Niche	Niche	1.16%	1e-04 ***
		Niche : Host Order	0.61%	0.0916 .
19	D_{5_mobile} ~Niche*Host Order	Niche	50.12%	1e-04 ***
		Host Order	8.40%	1e-04 ***
		Niche : Host Order	0.61%	0.0995 .
20	D_{5_mobile} ~Niche*Host Order*GC%	GC%	5.28%	1e-04 ***
		Total interaction terms	7.05%	
		Residuals	29.15%	
21	D_{5_mobile} ~Niche*Host Order*Family*GC%	GC%	3.56%	1e-04 ***
		Total interaction terms	10.90%	
		Residuals	10.71%	
22	D_{5_mobile} ~Niche*Host Order* GC%*Family	Family	14.59%	1e-04 ***
		Total interaction terms	10.90%	
		Residuals	10.71%	

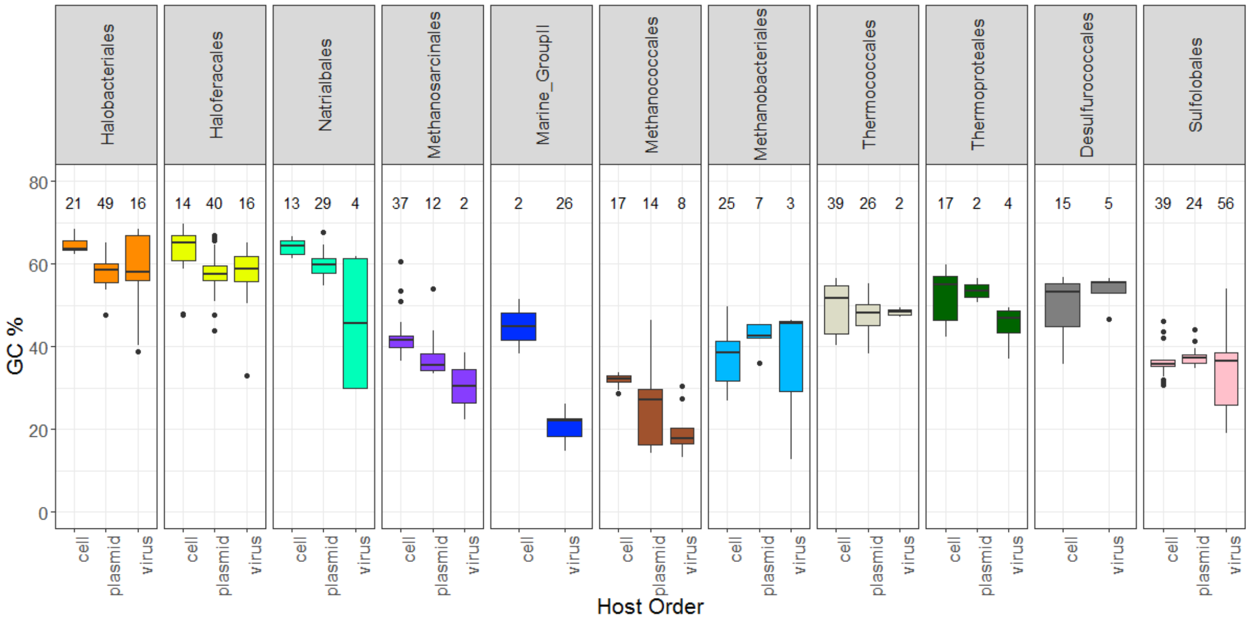

Additional File 04: Overview of the genomic GC contents across the dataset, according to the host order and to the type of element.

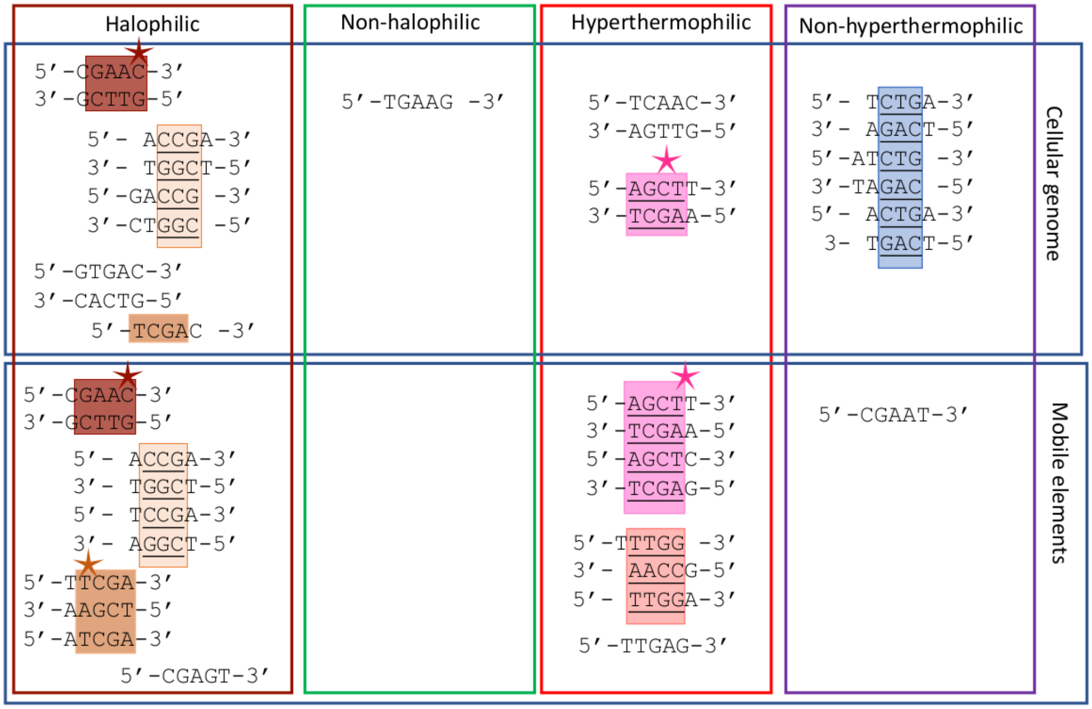

Additional file 05: Sets of 10 most discriminant 5-mers identified by PLS-DA. Reverse-complementary 5-mers and shared 3- or 4-mers are highlighted.

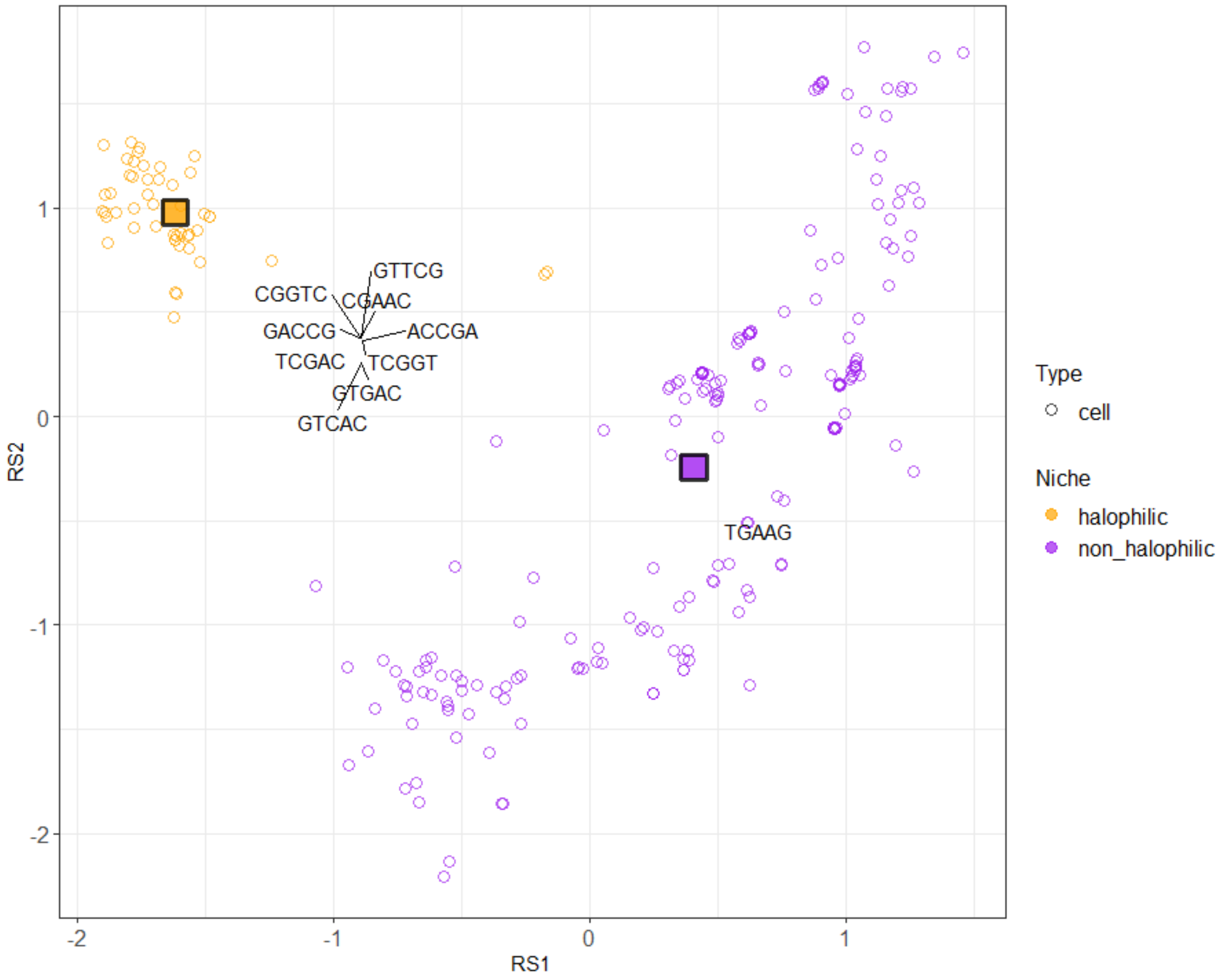

Additional file 06: PCA of the cellular genomes based on their 5-mer frequencies. Halophilic and non-halophilic archaea are highlighted. The projection of the 10 most discriminant 5-mers between the two groups, as identified by PLS-DA, is represented on the plot. Axes 1 and 2 preserve the variance of the shown data as best possible.

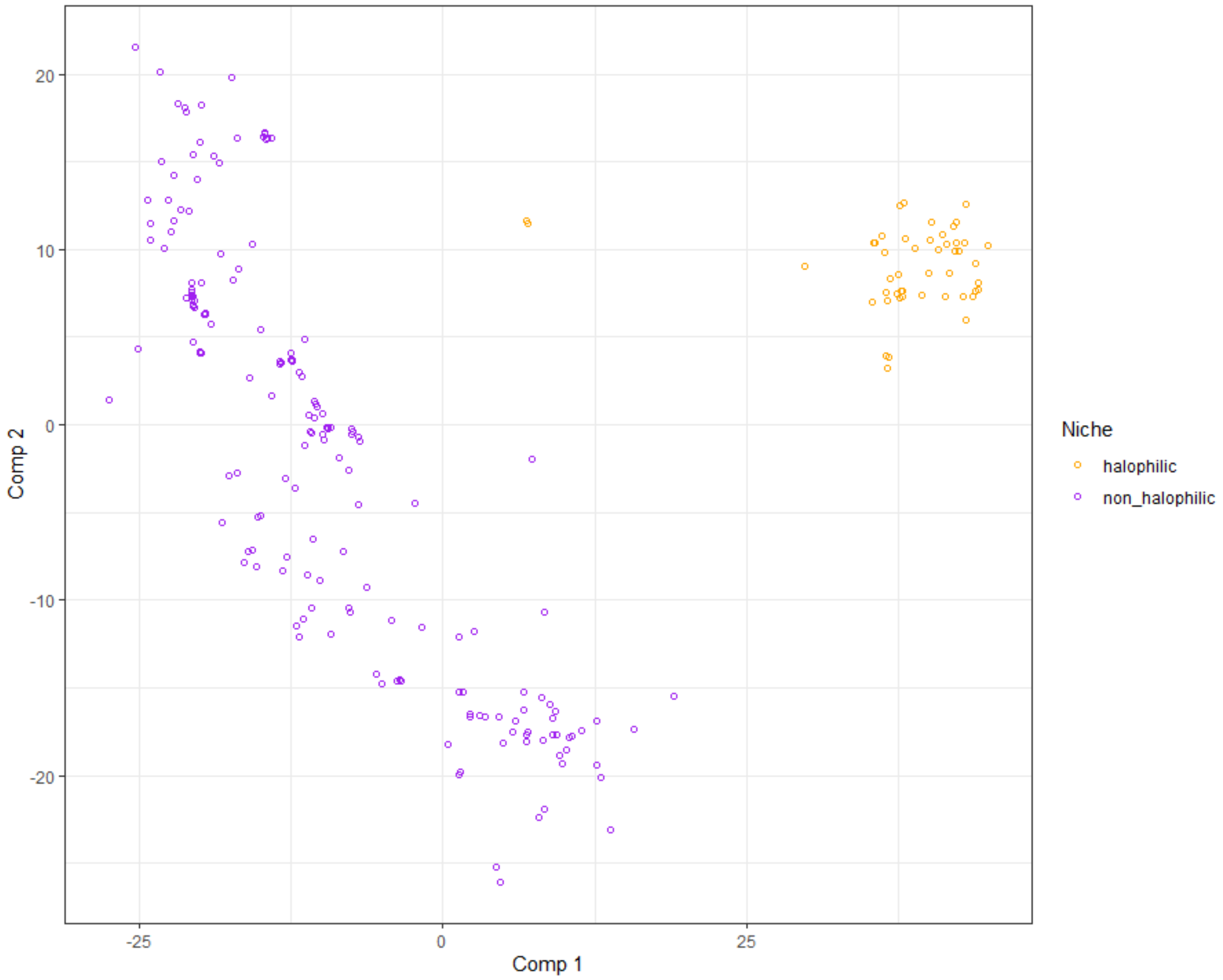

Additional file 07: PLS-DA of the cellular genomes based on their 5-mer frequencies, to identify discriminant 5-mers between halophilic and nonhalophilic archaea. Components 1 and 2 are the axes which separate the halophiles from the nonhalophiles as best possible.

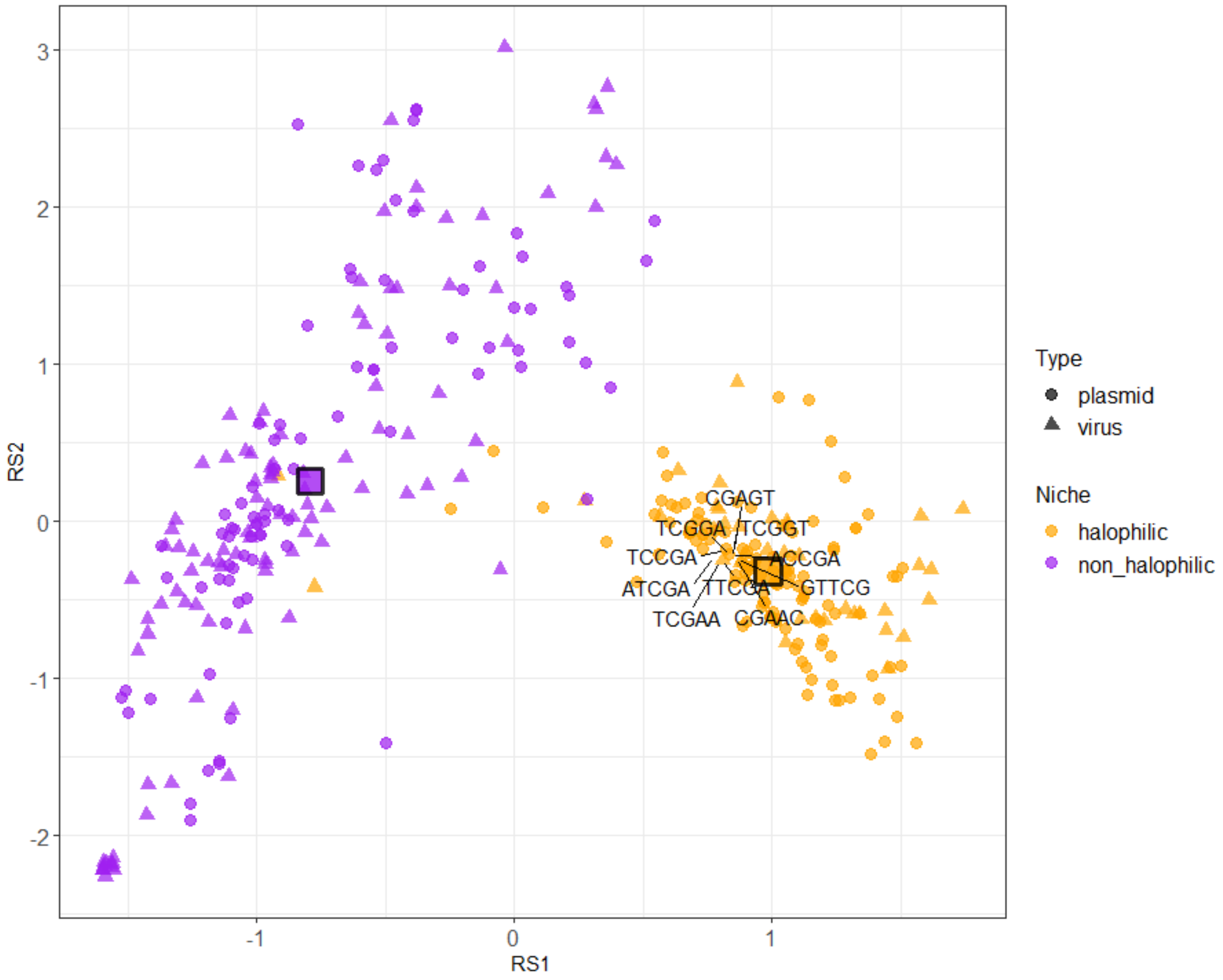

Additional file 08: PCA of the viral and plasmid genomes based on their 5-mer frequencies. Halophilic and nonhalophilic elements are highlighted. The projection of the 10 most discriminant 5-mers between the two groups, as identified by PLS-DA, is represented on the plot. Axes 1 and 2 preserve the variance of the shown data as best possible.

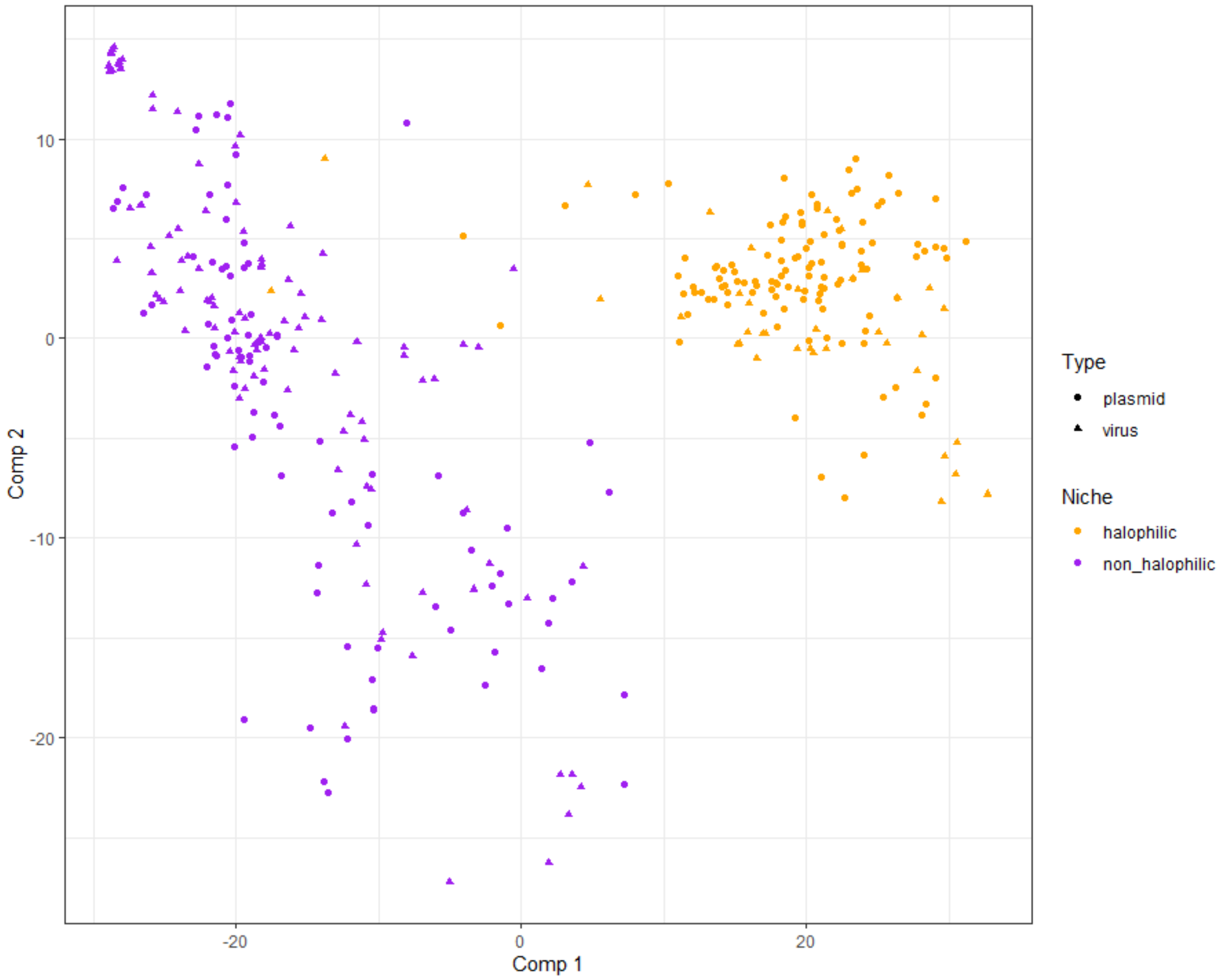

Additional file 09: PLS-DA of the viral and plasmid genomes based on their 5-mer frequencies, to identify discriminant 5-mers between halophilic and nonhalophilic archaeal mobile elements. Components 1 and 2 are the axes which separate the halophiles from the nonhalophiles as best possible.

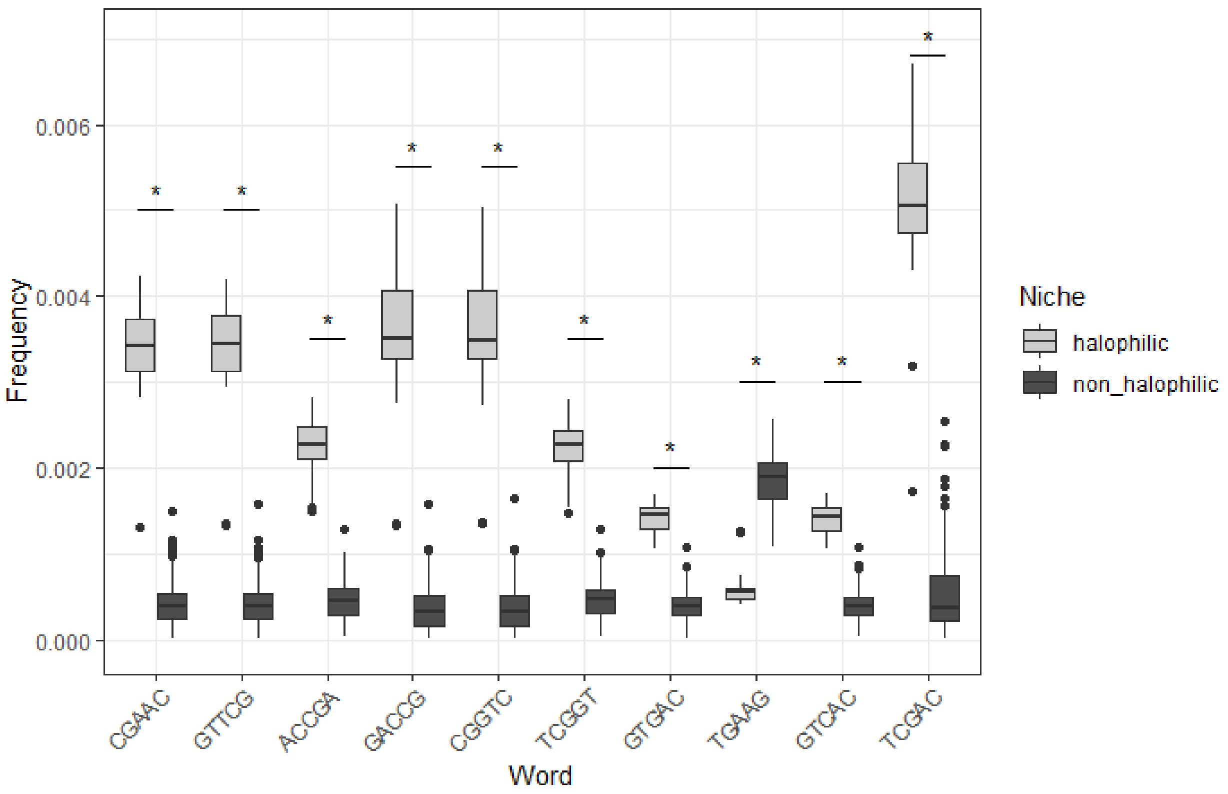

Additional file 10: Boxplots of 5-mer frequencies for the 10 most discriminant 5-mers between halophilic and nonhalophilic archaeal cellular genomes. The stars indicate a statically significant difference between the two groups (Mann-Whitney-Wilcoxon test, $p \leq 0.01$).

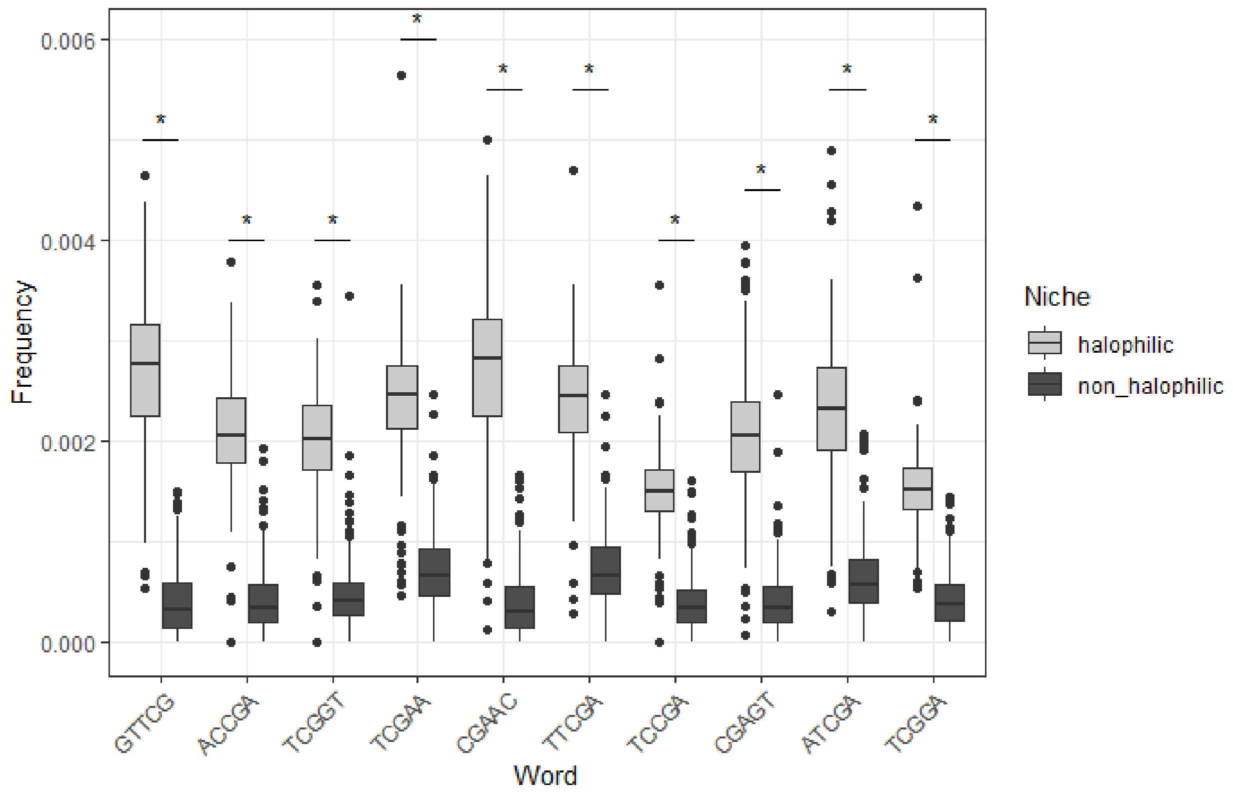

Additional file 11: Boxplots of 5-mer frequencies for the 10 most discriminant 5-mers between halophilic and nonhalophilic archaeal viral and plasmid genomes. The stars indicate a statically significant difference between the two groups (Mann-Whitney-Wilcoxon test, $p \leq 0.01$).

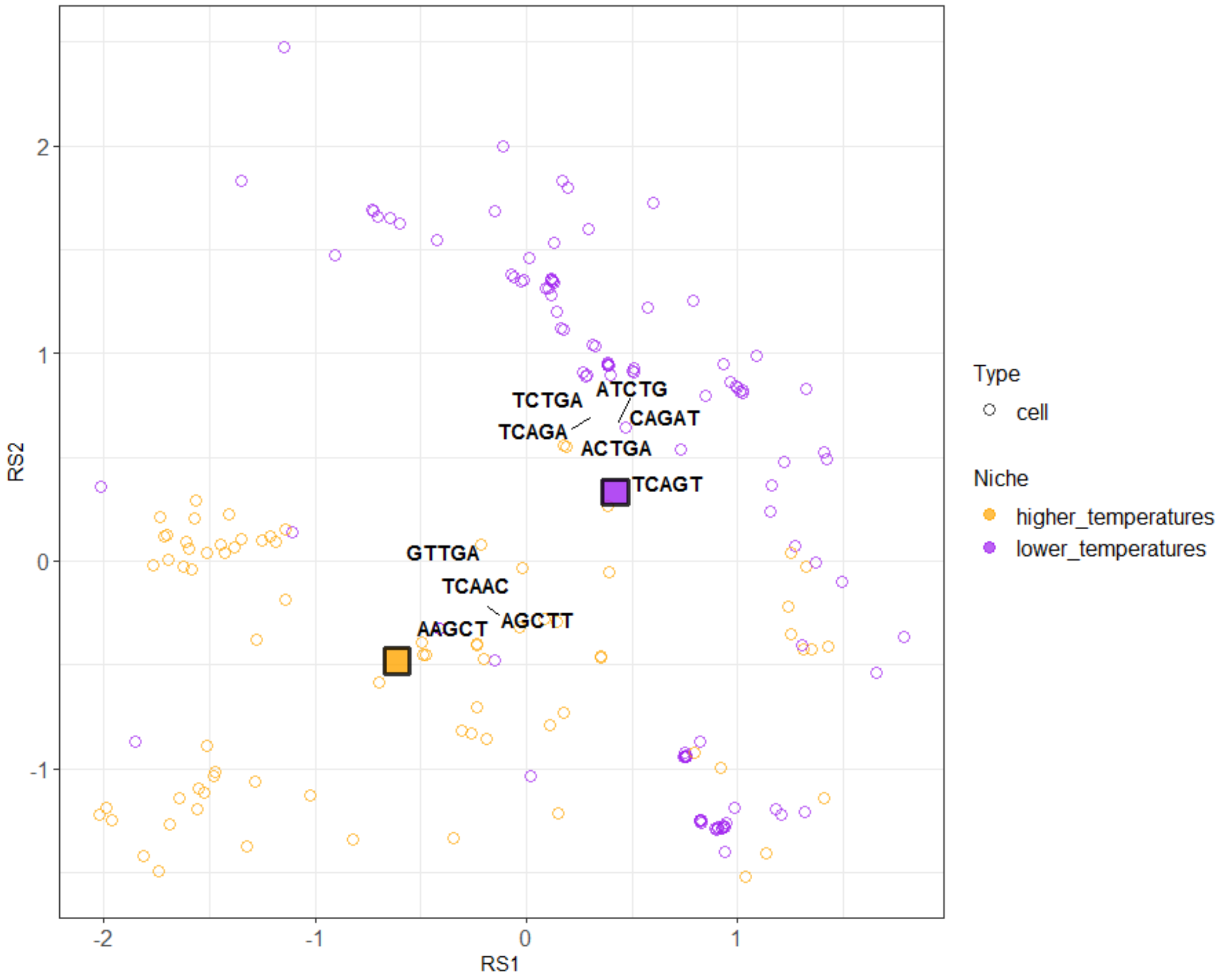

Additional file 12: PCA of the nonhalophilic cellular genomes based on their 5-mer frequencies. Individuals are colored according to their growth temperature. Lower temperature: <80°C; Higher temperature: ≥ 80°C. The projection of the 10 most discriminant 5-mers between the two groups, as identified by PLS-DA, is represented on the plot. Axes 1 and 2 preserve the variance of the shown data as best possible.

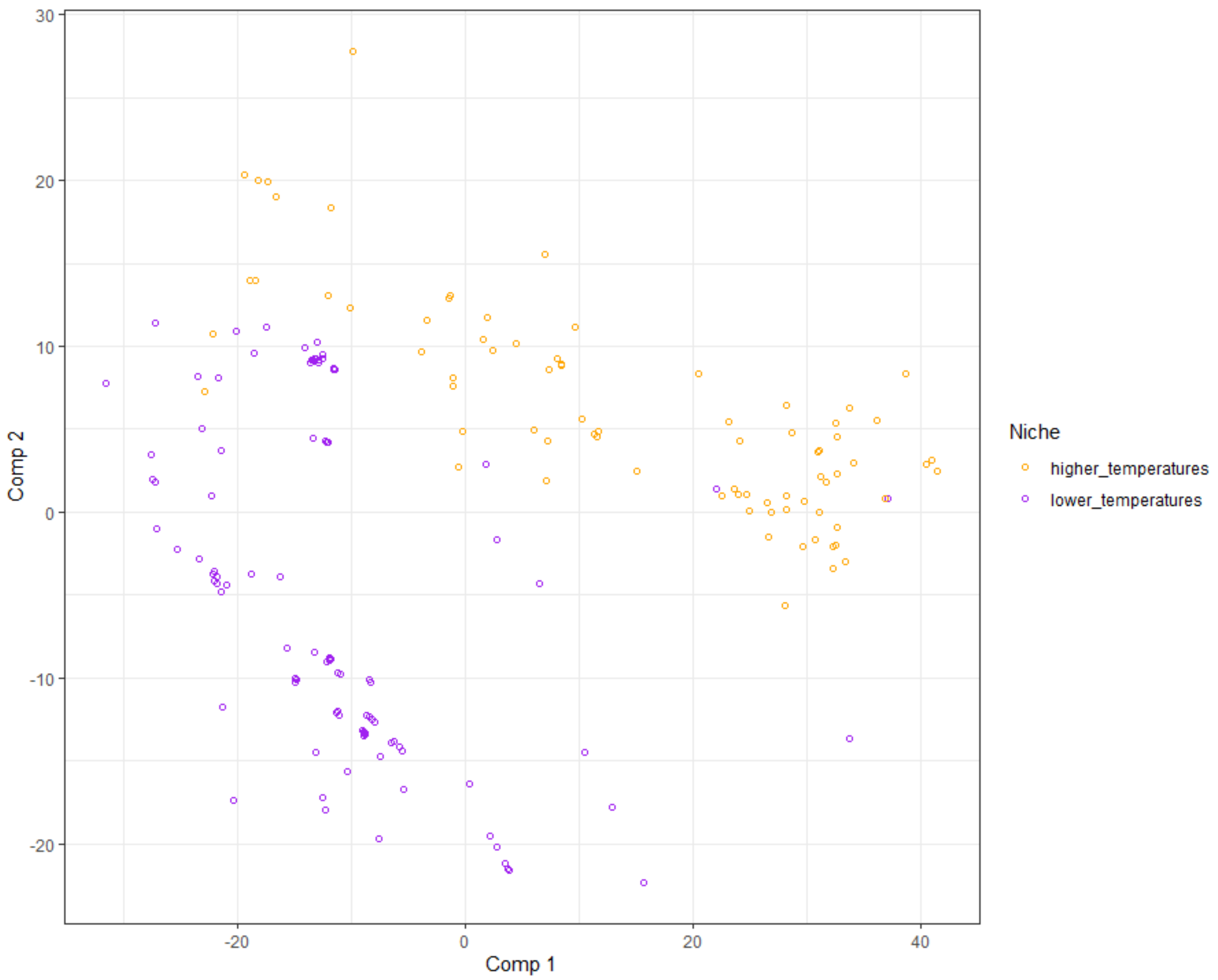

Additional file 13: PLS-DA of the nonhalophilic cellular genomes based on their 5-mer frequencies, to identify discriminant 5-mers between high growth temperature (≥ 80°C) and low growth temperature (<80°C) archaea. Components 1 and 2 are the axes which separate the high growth temperature from the low growth temperature archaea as best possible.

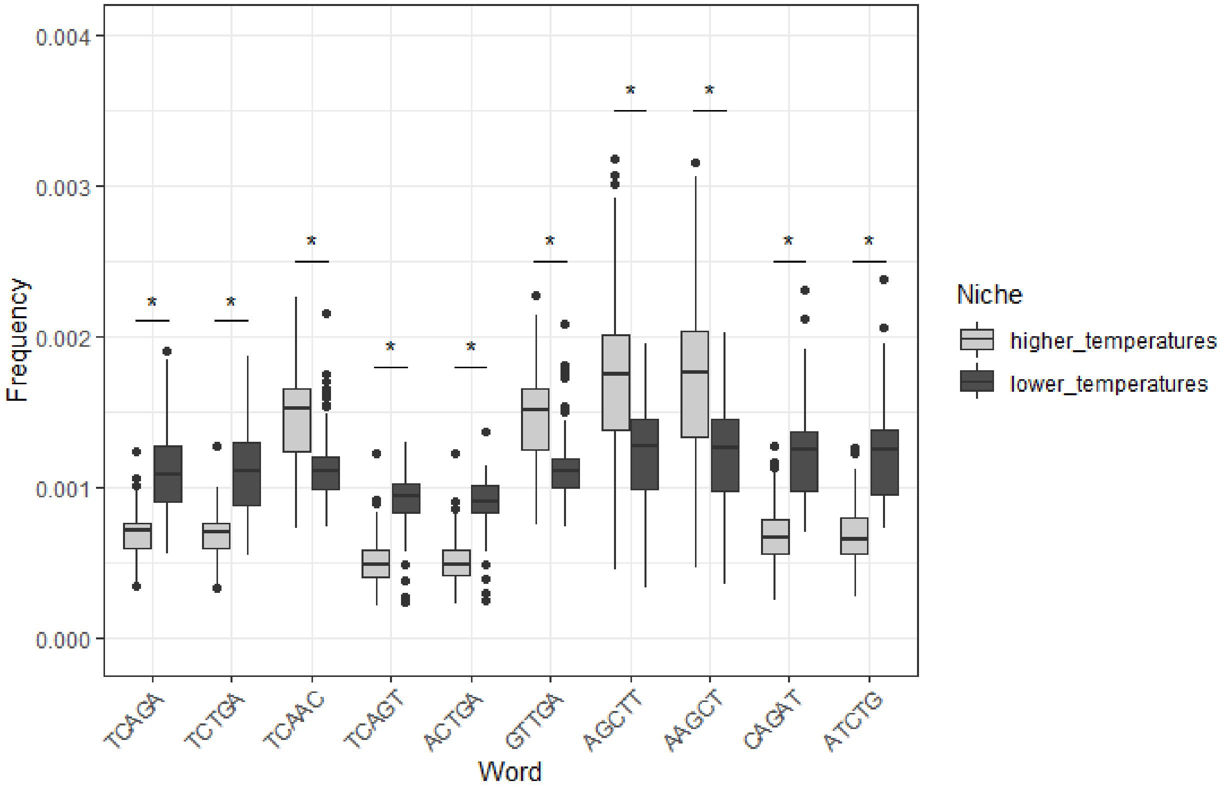

Additional file 14: Boxplots of 5-mer frequencies for the 10 most discriminant 5-mers between cells with higher growth temperatures (≥80°C) and lower growth temperatures (<80°C), among non-halophilic archaea. The stars indicate a statically significant difference between the two groups (Mann-Whitney-Wilcoxon test, $p \leq 0.01$).

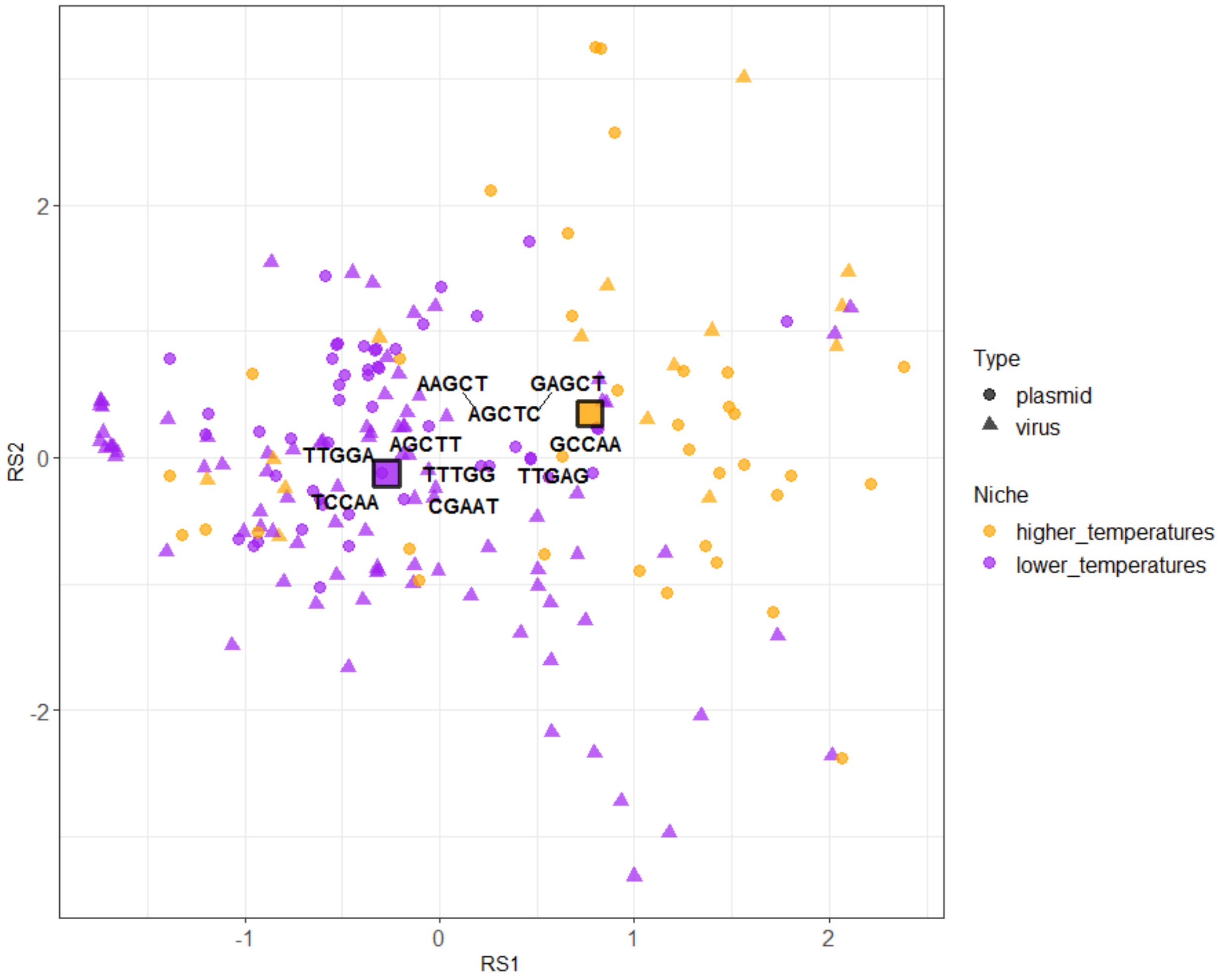

Additional file 15: PCA of viral and plasmid genomes from nonhalophilic archaeal hosts, based on their 5-mer frequencies. Higher host growth temperatures (≥80°C) and lower host growth temperatures (<80°C) are highlighted. The projection of the 10 most discriminant 5-mers between the two groups, as identified by PLS-DA, is represented on the plot. Axes 1 and 2 preserve the variance of the shown data as best possible.

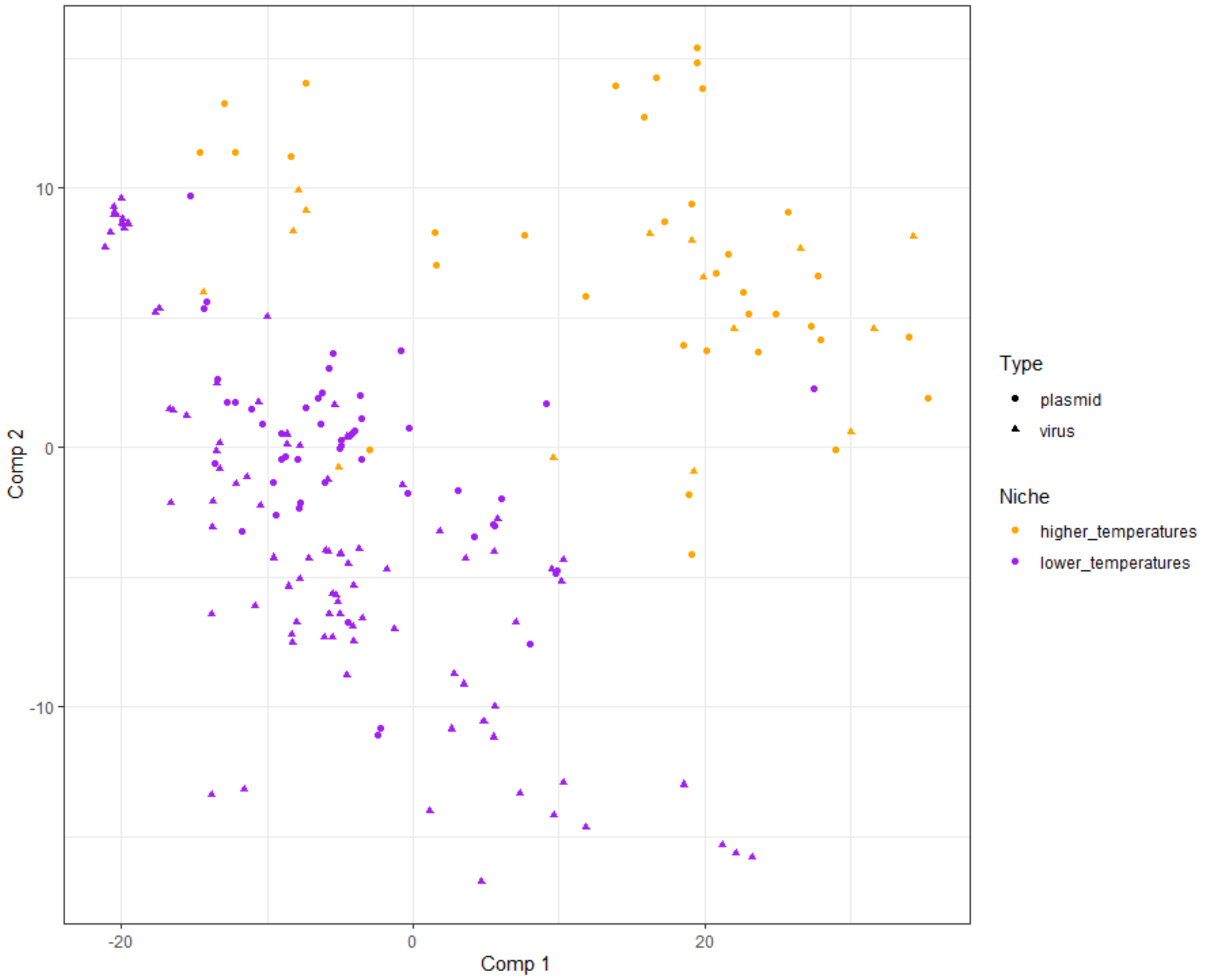

Additional file 16: PLS-DA of the viral and plasmid genomes from nonhalophilic archaeal hosts, based on their 5-mer frequencies, to identify discriminant 5-mers between archaeal mobile elements with high (≥ 80°C) and low (<80°C) host growth temperature. Components 1 and 2 are the axes which separate as best possiblethe high host growth temperature archaeal mobile elements from the low host growth temperature ones.

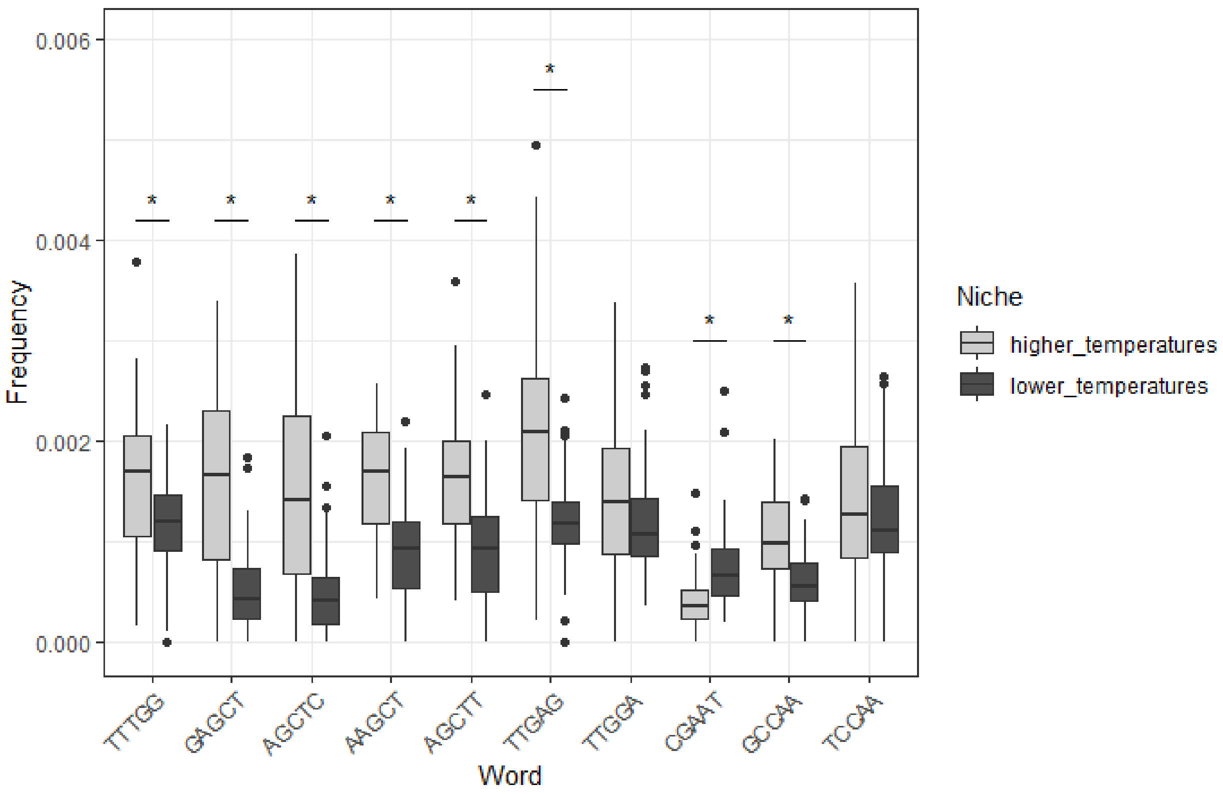

Additional file 17: Boxplots of ten 5-mer frequencies in extrachromosomal elements from nonhalophilic archaeal hosts, according to the growth temperature of their host. These 5-mers are the 10 most discriminant ones among nonhalophilic archaeal cells, according to their growth temperature, as identified by PLS. Higher growth temperatures: ≥80°C; lower growth temperatures: <80°C. The stars indicate a statically significant difference between the two groups (Mann-Whitney-Wilcoxon test, $p \leq 0.01$).

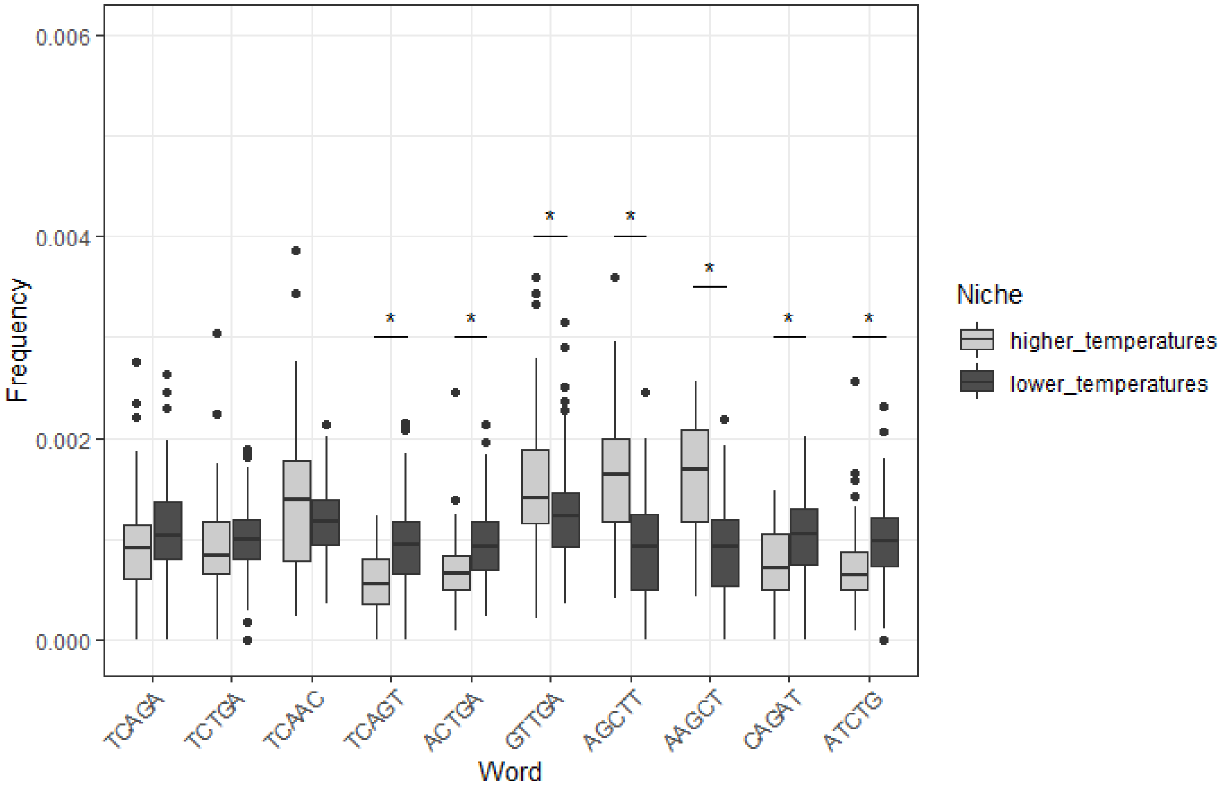

Additional file 18: Boxplots of 5-mer frequencies for the 10 most discriminant 5-mers among extrachromosomal elements from non-halophilic archaeal hosts, according to the growth temperature of their host. Higher growth temperatures: ≥80°C; lower growth temperatures: <80°C. The stars indicate a statically significant difference between the two groups (Mann-Whitney-Wilcoxon test, $p \leq 0.01$).

Additional File 19. Synthetic overview of the number of genomes included in the study.

	Cells	Conjugative plasmids	Other plasmids	Viruses	Total
Crenarchaeota	***71***	***13***	***13***	***65***	***162***
Desulfurococcales	15			5	**20**
Sulfolobales	39	13	11	56	**119**
Thermoproteales	17		2	4	**23**
Euryarchaeota	***173***	***12***	***165***	***77***	***427***
Halobacteriales	25	10	39	16	**90**
Haloferacales	15	2	38	16	**71**
Natrialbales	13		29	4	**46**
Methanosarcinales	37		12	2	**51**
Marine_GroupII	2			26	**28**
Methanobacteriales	25		7	3	**35**
Methanococcales	17		14	8	**39**
Thermococcales	39		26	2	**67**
**Total**	**244**	**26**	**178**	**142**	**589**

Additional File 20. PERMANOVA analyses of the 5-mer profiles from *Halobacteria* extrachromosomal elements

Model	Factor	Explained variance	Statistical significance
$D_{5\_mobile\_halo}$ ~GC%	GC%	14.17%	1e-04 ***
$D_{5\_mobile\_halo}$ ~Genome length	Genome length	2.10%	0.0039 **
$D_{5\_mobile\_halo}$ ~Type (virus / plasmid)	Type	1.99%	0.0046 **
$D_{5\_mobile\_halo}$ ~Type (virus / megaplasmid / big plasmid / plasmid	Type	6.49%	2e-04 ***
$D_{5\_mobile\_halo}$ ~Family	Family	30.04%	1e-04 ***
$D_{5\_mobile\_halo}$ ~Host order*Host genus	Host order	5.28%	0.0001 ***
	Host genus	17.81%	0.0338 *
$D_{5\_mobile\_halo}$ ~GC%*Family	GC%	14.17%	1e-04 ***
	Family	29.25%	1e-04 ***
	GC% : Family	12.10%	9e-04 ***
$D_{5\_mobile\_halo}$ ~Host genus*Family	Host genus	23.09%	1e-04 ***
	Family	25.62%	1e-04 ***
	Host genus : Family	16.77%	0.0139 *

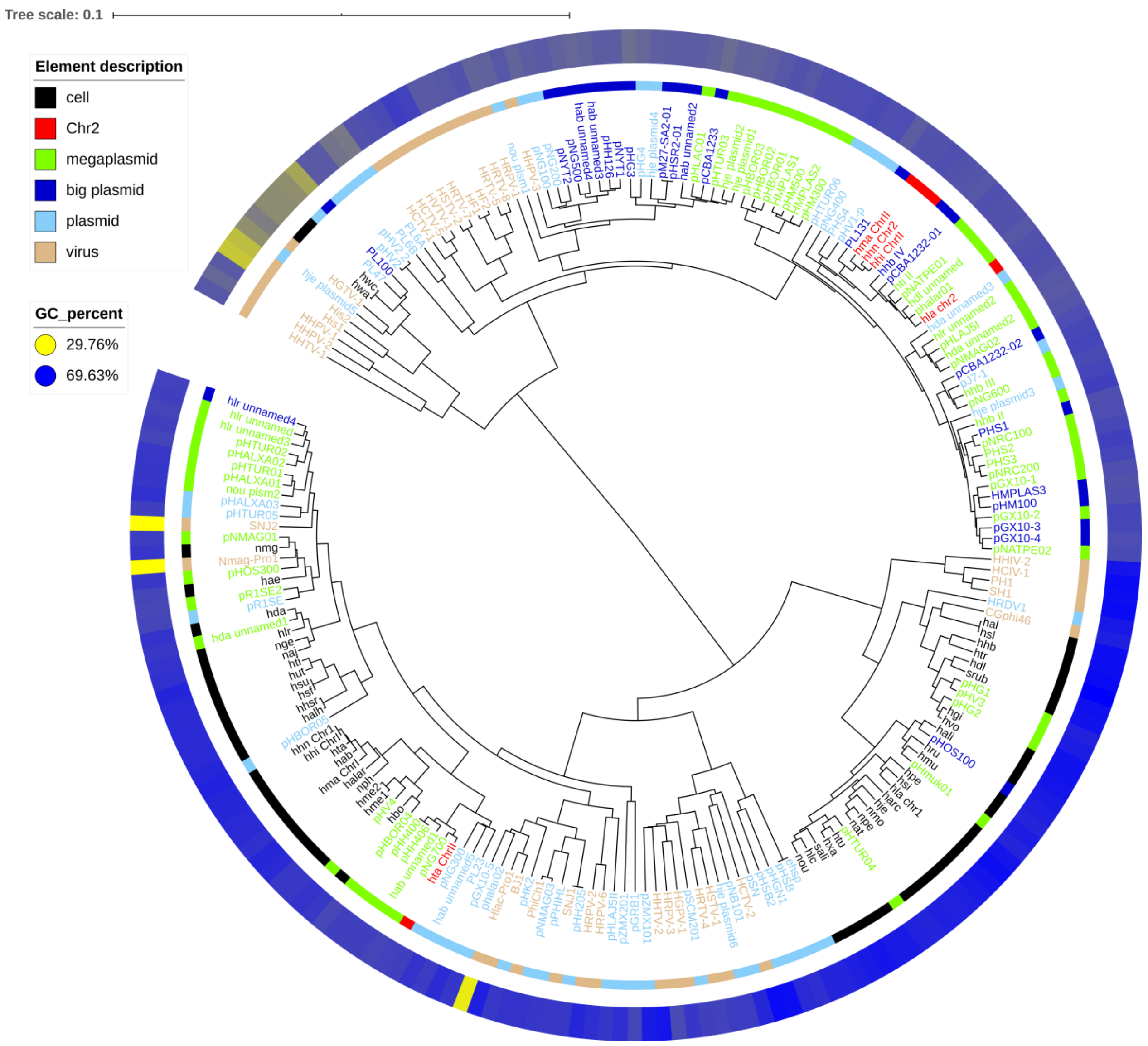

Additional file 21: Dendrogram of cells and extrachromosomal elements from halophilic archaea of the class *Halobacteria*, based on their genome 5-mer frequencies.

Additional File 22. Synthetic overview of the number of *Sulfolobales* genomes included in this study.

	Acidianus	*Metallosphaera*	*Stygiolobus*	*Sulfolobus*	undet. Sulfolobales	Total
cell	***4***	***8***		***27***		***39***
Sulfolobaceae	4	8		27		**39**
conjugative plasmid	***1***			***12***		***13***
pNOB8-like	1			12		**13**
other plasmid	***1***			***10***		***11***
pRN-like	1			10		**11**
virus	***15***	***2***	***1***	***34***	***4***	***56***
Ampullaviridae	3					**3**
Bicaudaviridae	2			2		**4**
Fuselloviridae	1			11	1	**13**
Lipothrixviridae	7			1		**8**
monocaudavirus				4		**4**
Ovaliviridae				1		**1**
Portogloboviridae				1		**1**
Rudiviridae	2		1	12	1	**16**
Turriviridae				2		**2**
Other viruses		2			2	**4**
**Total**	**21**	**10**	**1**	**83**	**4**	**119**

Additional File 23. PERMANOVA analyses of the 5-mer profiles from *Sulfolobales* extrachromosomal elements

Model	Factor	Explained variance	Statistical significance
$D_{5\_mobile\_sulfo}$ ~GC%	GC%	33.10%	1e-04 ***
$D_{5\_mobile\_sulfo}$ ~Type (virus / plasmid)	Type	4.96%	0.0072 **
$D_{5\_mobile\_sulfo}$ ~Type (virus / CJ plasmid/ other plasmid)	Type	9.06%	0.0024 **
$D_{5\_mobile\_sulfo}$ ~Family	Family	57.39%	1e-04 ***
$D_{5\_mobile\_sulfo}$ ~Host genus	Host genus	18.01%	1e-04 ***
$D_{5\_mobile\_sulfo}$ ~GC%*Family	GC%	33.10%	1e-04 ***
	Family	32.84%	1e-04 ***
	GC% : Family	11.74%	1e-04 ***
$D_{5\_mobile\_sulfo}$ ~Family*GC%	Family	57.39%	1e-04 ***
	GC%	8.54%	1e-04 ***
	Family : GC%	11.74%	1e-04 ***
$D_{5\_mobile\_sulfo}$ ~Host genus *Family	Host genus	18.01%	1e-04 ***
	Family	50.67%	1e-04 ***
	Host genus : Family	7.08%	2e-04 ***

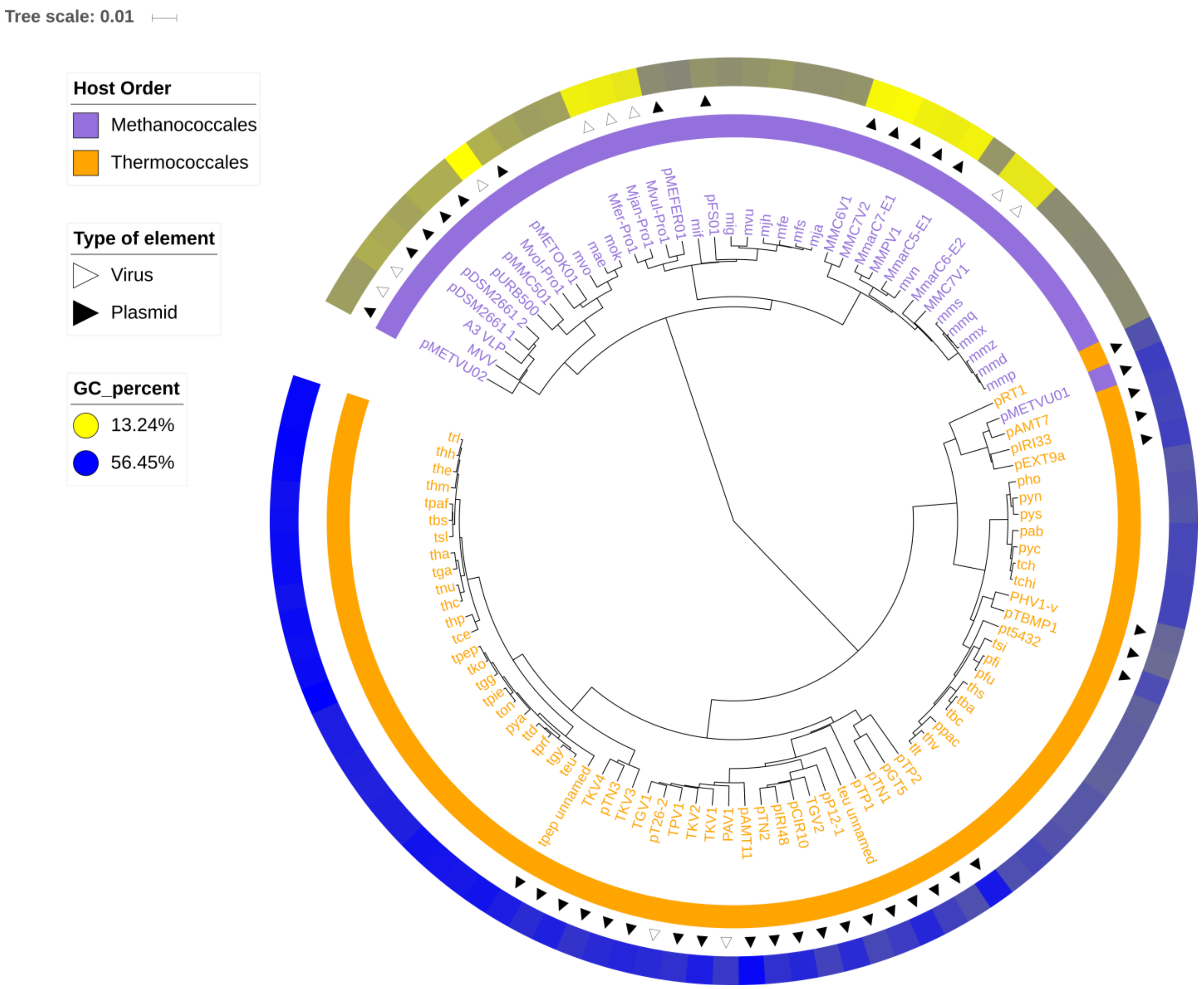

Additional file 24: Dendrogram of cells and extrachromosomal elements from the orders *Methanococcales* and *Thermococcales*, based on their genome 5-mer frequencies.

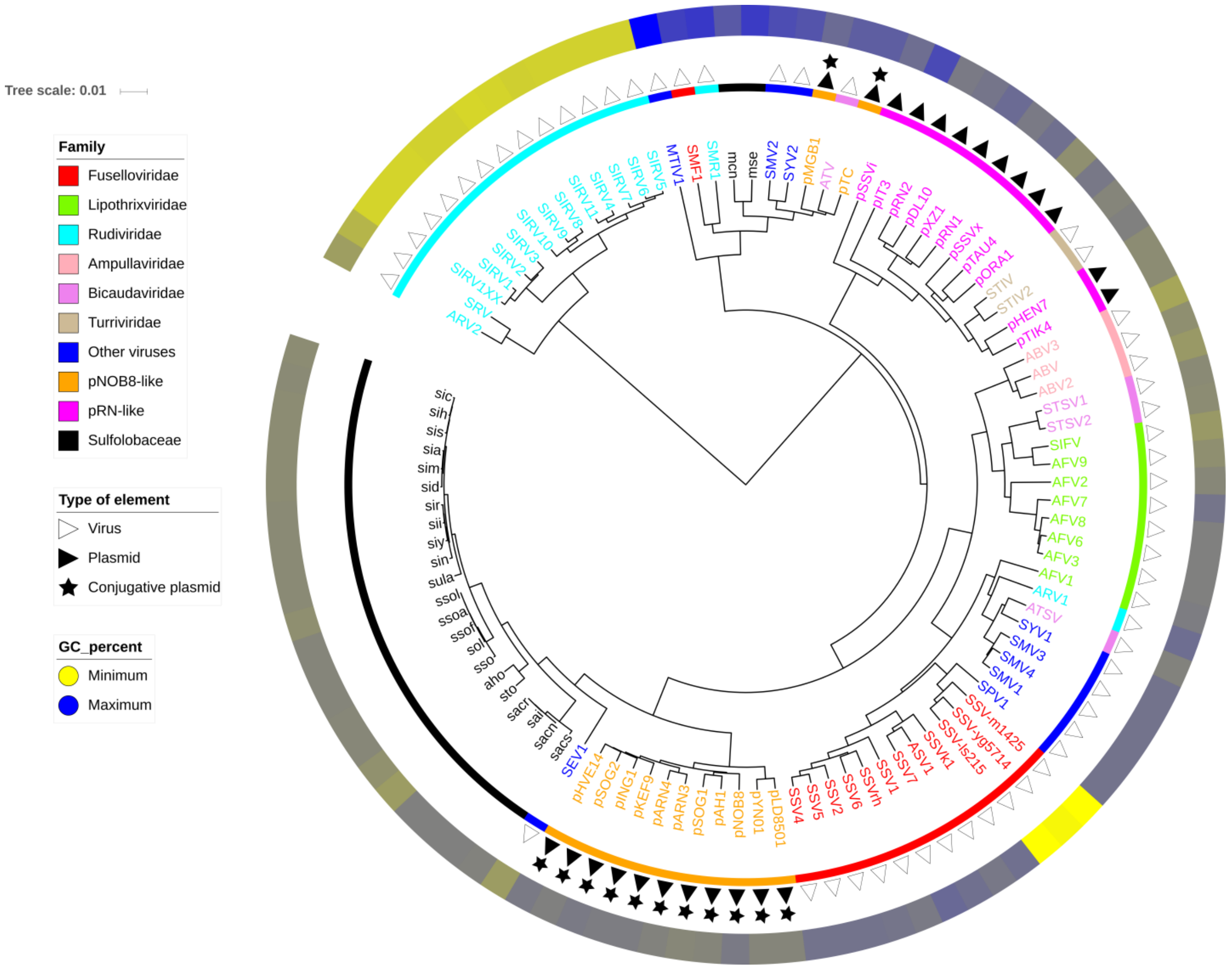

Additional file 25: Dendrogram of cells and extrachromosomal elements from the order *Sulfolobales*, based on their genome 5-mer frequencies, for a previous version of the dataset.

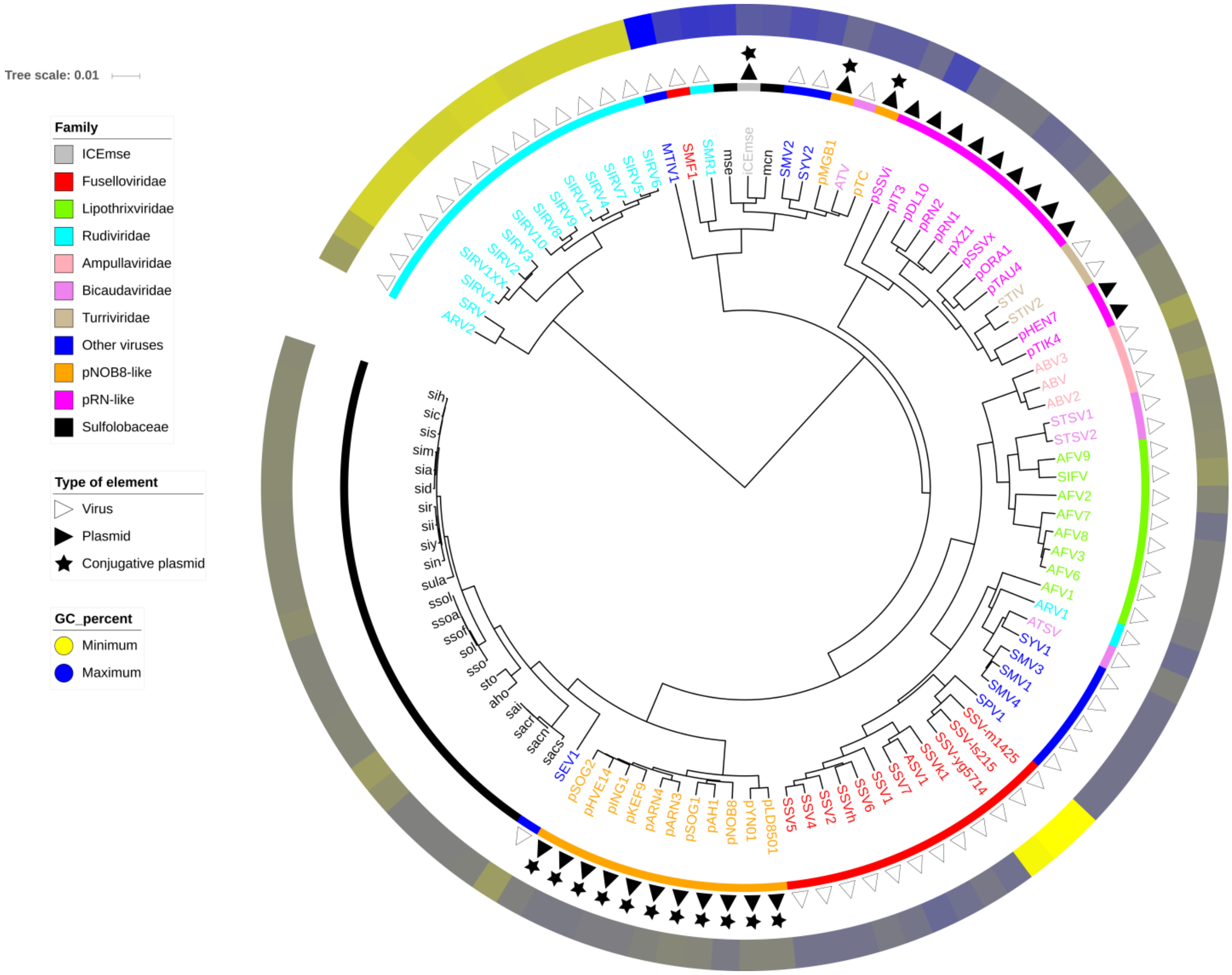

Additional file 26: Dendrogram of cells and extrachromosomal elements from the order *Sulfolobales*, based on their genome 5-mer frequencies, for a previous version of the dataset, including iCEmse.

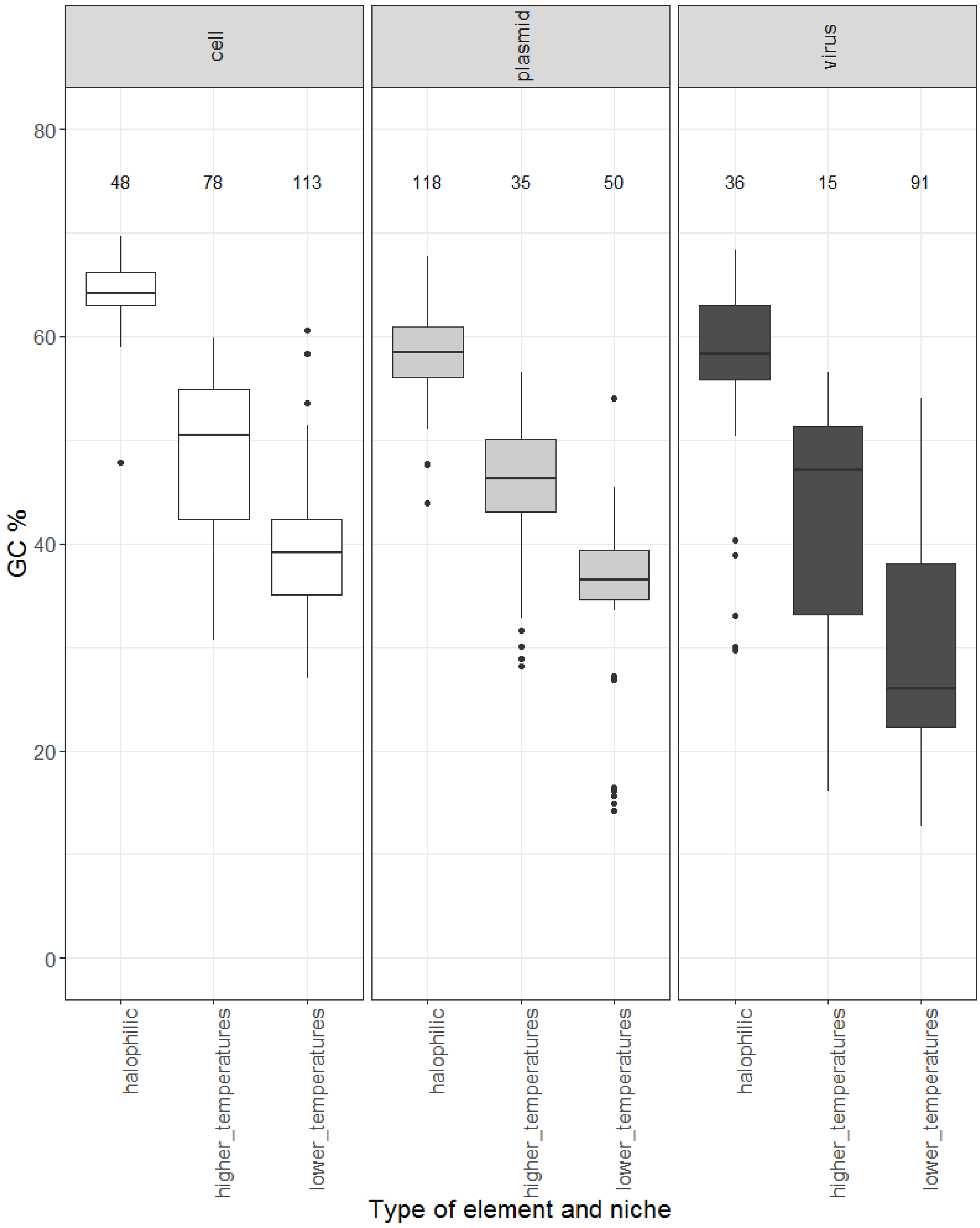

Additional file 27: Overview of the genomic GC contents across the dataset, according to the type of element and ecological niche.

Additional File 28. ANOVA of the genomic GC percent values from the complete dataset

Model	Factor	Explained variance	Statistical significance
GC%~Genome length	Genome length	1.00%	0.0156 *
GC%~Type (cell, virus, plasmid)	Type	12.98%	<2e-16 ***
GC%~Niche	Niche	67.15%	<2e-16 ***
GC%~Host Order	Host Order	78.27%	<2e-16 ***
GC%~Genus (host) or Family (extrachromosomal element)	Genus or family	89.40%	<2e-16 ***
GC%~Host Phylum*Host Order*Host Genus	Host Phylum	7.88%	<2e-16 ***
	Host Order	70.39%	<2e-16 ***
	Host Genus	7.13%	<2e-16 ***
GC%~Type*Host Order	Type	12.98%	<2e-16 ***
	Host Order	67.34%	<2e-16 ***
	Type : Host Order	2.71%	2.48e-10 ***
GC%~Host Order*Type	Host Order	78.27%	<2e-16 ***
	Type	2.05%	2.11e-14 ***
	Type : Host Order	2.71%	2.48e-10 ***
GC%~Genus or Family*Host Order	Genus or Family	89.39%	<2e-16 ***
	Host Order	2.99%	<2e-16 ***
	Genus or Family : Host Order	1.37%	4.03e-14 ***
GC%~Host Order*Genus or Family	Host Order	78.27%	<2e-16 ***
	Genus or Family	14.13%	<2e-16 ***
	Genus or Family : Host Order	1.37%	4.03e-14 ***
GC%~Niche*Host Order	Niche	67.15%	<2e-16 ***
	Host Order	12.56%	<2e-16 ***
	Niche : Host Order	1.05%	0.00162 **
GC%~Host Order *Niche	Host Order	78.27%	<2e-16 ***
	Niche	1.44%	3.91e-07 ***
	Niche : Host Order	1.05%	0.00162 **

Additional File 29. Dataset presentation.

The numbers of cellular genomes were overall well-balanced across the taxonomic orders, ranging from 13 for *Natrialbales* to 39 for *Sulfolobales* or *Thermococcales*. However, only 2 cellular genome sequences were available for the Marine group II archaea.

All the crenarchaea considered here are acidothermophiles (*Sulfolobales* order) or hyperthermophiles (*Desulfurococcales* and *Thermoproteales* orders) and they totalized 26 associated plasmid sequences as well as 65 viral sequences. For the euryarchaea, the *Halobacteria* class was the most represented. It consists exclusively of halophiles and encompasses 3 orders: *Halobacteriales*, *Haloferacales* and *Natrialbales*. In the dataset, *Halobacteria* members were associated to 118 plasmid sequences and 36 viral sequences. The methanogens are polyphyletic and divided into 6 orders at least [50]. The present dataset covered two class I methanogen orders, namely, *Methanobacteriales* and *Methanococcales*, as well as one class I methanogen order, namely, *Methanosarcinales*. Those methanogens were associated to 33 plasmid sequences and 13 viral sequences. For euryarchaea, we also included Marine Group II archaea, with 26 viral sequences. These archaea are abundant in oxygenated surface and deep marine waters [51] and they are stilluncultured. All their viral sequences were retrieved from metagenomic data [52]. Finally, a single order of hyperthermophilic euryarchaeota was also included, *Thermococcales*, with 26 plasmid sequences and 2 viral sequences.
